# Network Embedding Across Multiple Tissues and Data Modalities Elucidates the Context of Host Factors Important for COVID-19 Infection

**DOI:** 10.3389/fgene.2022.909714

**Published:** 2022-07-08

**Authors:** Yue Hu, Ghalia Rehawi, Lambert Moyon, Nathalie Gerstner, Christoph Ogris, Janine Knauer-Arloth, Florian Bittner, Annalisa Marsico, Nikola S. Mueller

**Affiliations:** ^1^ Computational Health Department, Helmholtz Center Munich, Neuherberg, Germany; ^2^ Informatics 12 Chair of Bioinformatics, Technical University Munich, Garching, Germany; ^3^ Translational Research in Psychiatry, MaxPlanck Institute of Psychiatry, Munich, Germany; ^4^ knowing01 GmbH, Munich, Germany

**Keywords:** multi-omic integration, network inference, network embedding, COVID-19, machine learning, polygenic risk score (PRS)

## Abstract

COVID-19 is a heterogeneous disease caused by SARS-CoV-2. Aside from infections of the lungs, the disease can spread throughout the body and damage many other tissues, leading to multiorgan failure in severe cases. The highly variable symptom severity is influenced by genetic predispositions and preexisting diseases which have not been investigated in a large-scale multimodal manner. We present a holistic analysis framework, setting previously reported COVID-19 genes in context with prepandemic data, such as gene expression patterns across multiple tissues, polygenetic predispositions, and patient diseases, which are putative comorbidities of COVID-19. First, we generate a multimodal network using the prior-based network inference method KiMONo. We then embed the network to generate a meaningful lower-dimensional representation of the data. The input data are obtained *via* the Genotype-Tissue Expression project (GTEx), containing expression data from a range of tissues with genomic and phenotypic information of over 900 patients and 50 tissues. The generated network consists of nodes, that is, genes and polygenic risk scores (PRS) for several diseases/phenotypes, as well as for COVID-19 severity and hospitalization, and links between them if they are statistically associated in a regularized linear model by feature selection. Applying network embedding on the generated multimodal network allows us to perform efficient network analysis by identifying nodes close by in a lower-dimensional space that correspond to entities which are statistically linked. By determining the similarity between COVID-19 genes and other nodes through embedding, we identify disease associations to tissues, like the brain and gut. We also find strong associations between COVID-19 genes and various diseases such as ischemic heart disease, cerebrovascular disease, and hypertension. Moreover, we find evidence linking PTPN6 to a range of comorbidities along with the genetic predisposition of COVID-19, suggesting that this kinase is a central player in severe cases of COVID-19. In conclusion, our holistic network inference coupled with network embedding of multimodal data enables the contextualization of COVID-19-associated genes with respect to tissues, disease states, and genetic risk factors. Such contextualization can be exploited to further elucidate the biological importance of known and novel genes for severity of the disease in patients.

## Introduction

The coronavirus strain severe acute respiratory syndrome coronavirus 2 (SARS-CoV-2) causes COVID-19, a respiratory illness, and is solely responsible for one of the deadliest pandemics in modern human history. Once infected, most patients experience symptoms such as cough, sore throat, fever, shortness of breath, nausea, and diarrhea. In severe cases, the disease leads to acute respiratory distress syndrome, a serious lung condition resulting in low blood oxygen (Tay et al., 2020). Even though the virus mainly affects the respiratory systems, a viral load has been found in many other tissues (Demichev, 2021). Hence, it is not surprising that studies demonstrated the effect of COVID-19 onto a wide range of systems, including cardiovascular, renal, hepatobiliary, and neurological systems (Gupta et al., 2020). These findings were recently underpinned by linking fatal COVID-19 cases to kidney and liver failure, also pointing out the key role of several chronic diseases in mortality of patients (Elezkurtaj et al., 2021). Among the most reported are arterial hypertension, obesity, ischemic heart disease, cerebrovascular disease, alcohol and nicotine abuse, and chronic obstructive pulmonary disease (COPD) (Elezkurtaj et al., 2021). Mortality rates were associated with lung damage initiated by a SARS-CoV-2 infection but powerfully predisposed by preexisting diseases (comorbidities).

Another potential contributor to disease pathogenesis is host genetics. Several genetic loci were shown to be associated with susceptibility to a severe disease course of COVID-19 (Ellinghaus et al., 2020). The genetic component was interrogated in a large international effort of the COVID-19 Host Genetics Initiative, which conducted genome-wide association studies (GWASs) and uncovered single-nucleotide polymorphisms (SNPs) that were correlated to severe cases of COVID-19 (The COVID-19 Host Genetics Initiative, 2020). Together, these studies revealed that the host antiviral defense mechanisms were related to genetic predisposition and that the disease affects different tissues and individuals in different ways, which are better understood in the context of human variety.

In addition to the GWAS studies, functional experimental assays have shed light on the molecular mechanisms of the response to SARS-CoV-2 infections in cell lines. Such studies investigated, for example, the interactome between the host and virus through ribonucleoprotein capture and immunoprecipitation (Gordon et al., 2020; Lee et al., 2020) to find host factors that can physically interact with viral proteins. Furthermore, CRISPR studies identified host factors critical for SARS-CoV-2 infection (Schneider et al., 2021; Wu et al., 2021). Another source for understanding the viral response comes from whole blood sample data, quantifying the genes, proteins, metabolites, and lipids differentially expressed in cases and controls (Shen et al., 2020; D’Alessandro et al., 2020; Di et al., 2020; Messner et al., 2020; Wu et al., 2021; Overmyer et al., 2021; Geyer et al., 2021; Demichev, 2021). Many efforts have been made to understand different aspects of the infection with SARS-CoV-2, yet an integrated view with multiple tissues is lacking. Montaldo et al. (2021) already recognized the importance of multi-omic studies to identify pathogenic mechanisms in COVID-19 development, which they carried out by a review of domain literature.

Methods for multi-omics data integration span from unsupervised multi-omic factor analysis (Argelaguet et al., 2018) over methods which maximize the correlation between multiple omics datasets (Singh et al., 2019) to multimodal network inference approaches (Ogris et al., 2021). In our previous work, we developed KiMONo, a versatile network inference tool (Ogris et al., 2021) that leverages prior information from existing biological networks to reduce the high-dimensional input space and model every gene measurement individually using a sparse group lasso. By aggregating selected features from KiMONo’s statistical models, a network consisting of different modalities can be generated, connecting the modeled genes with their explanatory variables. Such a multimodal network is, however, highly complex and difficult to analyze with classical network analysis tools, such as degree and betweenness analysis or module detection algorithms. To mine the network and extract meaningful associations, graph representation learning approaches have shown great promise when applied to analyze complex biomedical networks (Li et al., 2021; Nelson et al., 2019). The geometry of this embedding space is optimized to capture meaningful similarities or associations between nodes of a given network. It can be utilized to infer relationships between nodes in a network, for example, between genes and genetic risk score or genes and tissues and to understand the multimodal context for each factor of interest. The first efforts to prioritize important connected nodes have been conducted by GeneWalk (Ietswaart et al., 2021). Briefly, GeneWalk generates a low-dimensional embedding space of a gene–gene network together with their biological Gene Ontology terms by learning the relationships between nodes from random walks over the multimodal network. The authors showed that this low-dimension embedding from this unsupervised representation learning algorithm enabled a more informative characterization of each gene’s annotated terms with the underlying specific biological context.

Given the complexity of COVID-19 and the many genetic, general risk, and comorbidity factors that contribute to the different possible disease manifestations, we here aimed at deriving a multimodal view, especially from the genetic and comorbidity perspective, across the whole body. These multi-omic data were modeled into an embedding space for the efficient exploration of the relationship between modalities. However, data on COVID-19 including clinical phenotypes, genomic, and transcriptomic measures on a large scale and for different tissues of populations are still sparse, and therefore, cannot be fully exploited by multi-omics data integration methods to generate a global multi-tissue and cross-individual view of the disease. Thus, we leverage a population dataset prior to the COVID-19 outbreak that comprises comprehensive multi-tissue, multi-omics, and deep phenotyping data from the Genotype-Tissue Expression (GTEx) consortium (Carithers et al., 2015). In this study, we take an orthogonal approach to understand the complexity of symptoms, affected tissue, and individual genetic variation to the molecular response to COVID-19. To this end, we established a new analysis strategy by setting up a machine learning framework which combines network inference and embedding to integrate these pre-corona population data, uncover patterns in those data, and use this knowledge to understand the role of host factors important for COVID-19 in a broader context, in the light of other existing diseases, phenotypes, and genetic variation and gene expression across a broad range of tissues from GTEx.

First, we used the genomic information to calculate polygenic risk scores (PRSs) which reflected the genetic risk to develop a certain disease. For this, we used GWAS summary statistics from a range of diseases with associations to COVID-19, such as pneumological, cardiovascular, or metabolic diseases. In the next step, we integrated the PRS together with phenotypes and disease states (which can be viewed as comorbidities for COVID-19) and gene expression across GTEx tissues to generate a multimodal network using KiMONo. Finally, we applied a graph embedding approach, based on the DeepWalk algorithm (Perozzi et al., 2014), which uses shallow neural networks to learn an embedding of every node. This embedding representation summarizes the associations between nodes in the multimodal network into a single similarity value for each pair of nodes, allowing us to efficiently explore and interpret a complex network. Finally, we annotated genes in the embedding that were found in different experimental studies related to COVID-19, such as GWAS (The COVID-19 Host Genetics Initiative, 2020), CRISPR (Wei et al., 2021; Schneider et al., 2021), physical binding experiments (Gordon et al., 2020; Lee et al., 2020), and patient OMICS data from blood serum and plasma (Shen et al., 2020; D’Alessandro et al., 2020; Di et al., 2020; Messner et al., 2020; Wu et al., 2021; Overmyer et al., 2021; Geyer et al., 2021; Demichev, 2021). This allowed us to elucidate the associations of known COVID-19 genes to tissues, disease states, and genetic risk factors, which we call the multimodal context hereafter. Through our statistical framework for inferring and embedding multi-omic networks, we gained insights that go beyond classical network statistics and put known COVID-19 genes in a multimodal context.

## Material and Methods

In the following section, we present our two-step machine learning framework consisting of inference followed by embedding of a multimodal network. We evaluate the resulting embedding by investigating the proximity of tissue nodes to tissue-specific genes. Furthermore, we explore the embedding for a range of diseases and genetic predisposition of diseases. Finally, we overlay the literature-derived annotation of COVID-19 genes to the embedding and capture their multi-omic context.

### Genomic Data and Polygenic Risk Score Calculation

We used data from the GTEx consortium spanning 984 individuals, consisting of phenotypic information, gene expression, and genomic variation (SNPs). Polygenic risk scores (PRSs) represent the genetic load for developing a certain disease. For their calculation, GWAS summary statistics were obtained for a range of diseases including type II diabetes (T2D) and major depressive disorder (MDD) as well as three COVID-19 susceptibility, severity, and hospitalization studies (The COVID-19 Host Genetics Initiative, 2020). The full table with the GWAS study source can be found under Supplementary Table S1. Next, we lifted the individual-level genotype data, available for 866 individuals, from the reference genome GRCh38 to GRCh37/hg19 using the tool LiftOverPlink (Ritchie, 2014) to match the GWAS summary statistics. We ended up with 1,119,899 SNPs that were successfully mapped and used for the calculation of 27 PRSs. For polygenic risk score prediction, we used the PRS-CS tool (Ge et al., 2019), which implements a Bayesian regression approach and utilizes a continuous shrinkage (CS) on SNP effect sizes. To account for the correlation between SNPs in close proximity, the method uses an external linkage disequilibrium (LD) reference panel; in our case, we used the European LD reference panel constructed using the 1000 Genomes Project (Ge, 2018). The global shrinkage parameter phi, which is required for the adjustment of effect sizes and depends on the sparseness of the genetic architecture of a trait (Ge et al., 2019), was set based on each disease’s polygenicity and sample size as follows: (1) for polygenic traits with large GWAS sample sizes (≥250,000) the phi parameter was set to default, that is, its value was estimated from the data using a fully Bayesian approach; (2) traits with a number of samples less than 250,000 and with a number of significant SNPs (*p* ≤ 5e−08) less than or equal to 100 were considered having low polygenicity and thus phi was set to 1e−4; (3) traits with a number of samples less than 250,000 and with a number of significant SNPs (*p* ≤ 5e−08) larger than 100 were considered having high polygenicity and thus phi was set to 1e−2. In the final step, we used PLINK2 (Chang et al., 2015) (PLINK v2; Shaun and Chang, 2019) to calculate the overall risk of each individual in the GTEx cohort for different diseases and traits.

### GTEx Data Processing

In the GTEx consortium, gene expression was measured in a range of tissues and sub-tissues. Genes were filtered to keep only protein coding genes and excluding those on the chromosomes X, Y to reduce sex-specific effects, following previous studies (Melé et al., 2015; Saha et al., 2017). Mitochondrial genes were also excluded as they are under different transcriptional control and would require additional modeling. Next, low-expression genes (at least 0.1 TPM in 80% of samples) were filtered out and considered for further analysis only if they were included in the BioGrid protein–protein interaction database (Oughtred et al., 2019). In addition, samples were filtered out if the tissue of origin was related to the reproductive system such as ovary, uterus, prostate, and testis to minimize sex-specific biases and a low sample size *n* < 100. Of 56,200 genes initially present in the GTEx database, 7,251 genes passed the filtering process and 44 sub-tissue types from 30 tissues were used in the end (Supplementary Figure S1).

Technical covariates were available on tissue resolution, comprising the platform of sequencing, mode of sequencing (PCR based), PC genotyping components, and probabilistic estimation of expression residual (PEER) factors (Stegle et al., 2010) that account for confounding factors such as technical sequencing conditions. The authors performed a PCA to decompose data variation due to other causes, such as batch and genotyping components, accounting for the phylogenetic relationship between individuals. For the network inference, tissues were dummy-coded for the respective gene expression samples. For the reference level in the regularized linear models of sparse group lasso, we used the cultured fibroblasts samples as they are sufficiently distinct from all other tissue groups. Phenotypic information, comprising BMI, sex and age, and the disease states, including renal failure, ischemic heart disease, liver disease and *MDD*, was coded as binary vectors. Together, they made up the features used as input for the network inference algorithm KiMONo. In summary, a total of 13,486 samples from 793 individuals had the complete set of existing diseases (*n* = 12), phenotype (*n* = 3), gene expression (*n* = 7,251), tissue (*n* = 44), covariates (*n* = 78), and calculated PRS scores (*n* = 27).

### Network Inference and Embedding

To derive similarities between multimodal data, our two-step framework first infers a multimodal network and projects the nodes into a low-dimensional embedding space from which we compute similarities. For the generation of the multi-omic network, we used KiMONo to select features statistically contributing to the prediction of the expression pattern of each gene. The feature selection process applied by KiMONo works both on the modality groups (genes, phenotype, etc.) and on the individual features. The features retained by the sparse group lasso model from KiMONo were introduced in a network as nodes, linked to the node for the modeled gene.

$$y_{gene_{i}}∼\sum_{m\;\in \;modalities}\;\beta_{m}X_{m}.$$

For every gene *i*, we used direct interaction partner gene expression as additional predictors, which is a core concept of the KiMONo method. The BioGrid protein–protein interaction database with experimentally validated interactions was used as prior information to preselect gene–gene interactions to include a reduced number of genes to the sparse group lasso model. No prior information was used to filter the other modalities which resulted in having phenotypes, disease states (comorbidities of COVID-19), tissues, and phenotypic information as input to every gene model. To avoid statistical overrepresentation of edges between network nodes with no prior information applied, reverse models were calculated by modeling the values of the nongene features from all previously selected genes. Only the most influential genes ranked by their absolute beta (top 30%) were retained to harmonize the magnitude of edges between gene–gene and gene–nongene. We then assembled a multimodal network by connecting all modeled features with their explanatory variables, as identified by KiMONo models. Stability selection was performed over 30 runs, and features were retained if the feature was selected in more than 70% of the runs to only consider robustly selected features. Default filtering steps of *R* ^2^ > 0.01 and absolute mean beta coefficient >0.01 were applied on the inferred gene models to reduce noisy connections and ensure high-quality models.

The second step in our framework was to learn the low-dimensional embedding of the multimodal network by applying the GeneWalk embedding method (Ietswaart et al., 2021). Based on DeepWalk (Perozzi et al., 2014), the algorithm first generates sequences of nodes from unbiased random walks across the network. Then, a one hidden layer neural network learns to predict the target node based on the surrounding nodes in the random walk sequence following the SkipGram model (Mikolov et al., 2013; Mikolov et al., 2013). By varying the sliding window size, that is, the truncated length of the random walk, more or less large neighborhoods and direct or indirect neighboring node pairs are included. After training, the embeddings of each node can be extracted from the weights of the hidden layer of the shallow neural network. They can be used to determine its proximity to any other node in the embedding space by calculating the cosine similarity between the two embedding vectors. We refer to this value as the similarity between two nodes.

We applied a gridsearch on a smaller-sized network to determine optimal parameters for the algorithms. The network was calculated only on the data from brain samples, hereafter referred to as “brain network.” The parameters were “window size” = [2, 3] for the definition of positive examples and the “dimension of the embedding” = [4, 8, 16, 32] during the training process. These were tuned by maximizing the variance of the similarity distribution of 10,000 randomly sampled nodes. The highest variance reflects the highest information content of the network’s node in the embedding space without overfitting the data. The set of optimal embedding parameters used for all following analyses were window size = 2 and embedding dimension = 16. We then performed the embedding of the entire network 100 times to account for variability in the stochastic walk samples, yielding in 100 vector embeddings. In these, we analyzed the relationship between nodes that displayed the highest cosine similarity score to a given query node of interest, such as a disease or comorbidity node. Here, for each query node, we extracted the top 1,000 most similar nodes (according to the cosine similarity measure) for each of the 100 runs of the embedding. A node was considered robustly similar to a query node if it occurred in its top 1,000 in at least 80 of the 100 runs. Finally, for each query node, its associated robust nodes were ranked by their maximal similarity score. These sets of most similar nodes, thus, represented the multimodal contextualization of genes, which we used to elucidate the relationship between each of the COVID-19–associated genes and tissues or diseases.

Our machine learning framework was implemented in R and python and is freely available under https://github.com/cellmapslab/embed_multimodalNet.

### Tissue Enrichment Analysis

We expected genes which are preferentially expressed in a certain tissue or are tissue-specific to be closer in the embedding space to the node representing that tissue type compared to the nodes of other tissues. For the validation of the overall approach, we compared the *n* = 50, 100, 200, 300, and 500 topmost similar and least similar genes to tissue nodes according to their mean similarity score across 100 runs. Validation was performed using genes with tissue-enhanced expression from the protein atlas (Uhlén et al., 2015). For example, for the brain, we searched for “tissue_category_rna: brain; tissue enhanced AND sort_by: tissue specific score” on the web server (The Human Protein Atlas, 2022).

For this validation approach, we focused on the tissue brain and liver as these tissues had the largest number of samples and the highest amount of tissue-enhanced genes within the protein atlas. We computed the odds ratio of finding a tissue enhancement within the set of genes most similar compared to the set of genes least similar to that tissue. The raw expression within the GTEx dataset was visualized through a heatmap, and gene mRNA levels in the most-similar tissue were compared with the levels of other tissues to confirm their tissue specificity.

### COVID-19–Related Host Factors and Investigation of Multimodal Context

To study the multimodal context of COVID-19–associated genes, we compiled published SARS-CoV-2/COVID-19-related molecular datasets across four different types of experiments. We focused on tissues, disease states, and PRS in the proximity in the embedding space of these SARS-CoV-2/COVID-19–associated genes, proteins, and variants. In this case, specifically, instead of taking the top 1,000 nodes, we set a threshold on the similarity score (namely >0.65) to expand the similarity-based search space in order to include more nongene node embeddings. This is because node embeddings in very close proximity to a COVID-19 gene were embeddings of other gene nodes. When the threshold was surpassed, we represented the similarity of tissues, disease states, and PRS to the literature-derived genes as similarity-based graphs. In these graphs, we only included genes in close proximity to nodes of PRS COVID-19 susceptibility, severity, and hospitalization. The reasoning was to focus on the genetic component of the predisposition to COVID-19.

***COVID-19 genetics.*** Full summary statistics of COVID-19 GWAS (without 23andMe data, release June 6, 2021) were downloaded for the reference genome GRCh38. SNPs reported as significant with *p* < 1e−3 in comparison of very severe cases versus population (A1), hospitalization versus non-hospitalization (B1), and hospitalization versus population (B2) (The COVID-19 Host Genetics Initiative, 2020). Significant variants were overlapped with ENSEMBL gene version 101 (using knowing01 Explore software) to identify affected genes resulting in 515, 663, and 475 genes for A1, B1, and B2, respectively.

***Viral-host direct protein interactions.*** Physical interaction studies investigated the interactome between the host and SARS-CoV-2 virus using ribonucleoprotein captures and immunoprecipitation (Gordon et al., 2020; Lee et al., 2020). For the ribonucleoprotein captures, we used the 109 proteins that were regarded as the “SARS-CoV-2 RNA interactome” (Lee et al., 2020). For the immunoprecipitation experiment, we used the same high-confidence scoring criteria with MiST score ≥0.7, a SAINTexpress Bayesian false-discovery rate (BFDR) ≤0.05, and an average spectral count ≥2 (Gordon et al., 2020).

***CRISPR phenotype screens.*** The third set was built from genes from CRISPR studies that identified host factors critical for SARS-CoV-2 infection (Schneider et al., 2021; Stephenson et al., 2021).

The top 20 genes of pro-viral and anti-viral each were taken and ranked by the mean *z*-score in the Cas0-v2 conditions (Wei et al., 2021), and significant hits from Huh-7.5 37°C SARS-CoV-2 experiments were taken (Schneider et al., 2021).

***Patient multi-omics data***. We collected statistical results from eight studies. For all proteomics studies, we identified regulated proteins by applying a lax significance cutoff of adjusted *p* < 0.1 unless stated otherwise due to limited number of overall hits. From 262 in-patient sera, pairwise comparisons of the three time points of the first day of sampling, day of highest signal, and negatively tested for SARS-CoV-2 had been extracted (Geyer et al., 2021). From the sera of 19 individuals, pairwise comparisons of the three groups, healthy, non-severe, and severe COVID-19, had been extracted (Shen et al., 2020). From the sera of 38 individuals, we used the comparison controls versus patients with varying COVID-19 severities (D’Alessandro et al., 2020), to which we applied the lax filtering of *p* < 0.05 as the data lack multiple testing correction information. A total of 104 patient sera across different COVID-19 severities had been used to identify biomarkers, which were used without additional filtering (Messner et al., 2020). From a discovery cohort of 33 individuals, we extracted the published 90 differentially regulated proteins comparing control and COVID-19 patient sera on which no additional cutoff was applied (Di et al., 2020)*.* Blood plasma proteomics from 139 inpatients had been correlated with 86 diagnostic parameters and associated with severity using a lax-adjusted *p* < 0.1 cutoff (Demichev 2021). We additionally collected data from two multi-omic studies. Notably, we applied very stringent cutoff criteria on transcriptome data due to strong inflation of the number of regulated genes. From 231 COVID-19 patients without comorbidities, we extracted the pairwise comparisons of asymptomatic, mild, and severe cases using serum proteomics applying a lax-adjusted *p* cutoff <0.1 and a stringent cutoff on genes (adjusted *p* < 1e−10) measured by RNA-seq in whole blood (Wu et al., 2021). Finally, 128 individuals with and without COVID-19 infection were used to measure and compare association to disease state in context of ICU care by plasma proteomics (adjusted *p* < 0.1) and leukocyte transcriptomics (adjusted *p* <1e−10) as well as ICU × COVID-19 interaction analysis applying adjusted *p* < 0.1 for both omics (Overmyer et al., 2021).

## Results

**FIGURE 1:**
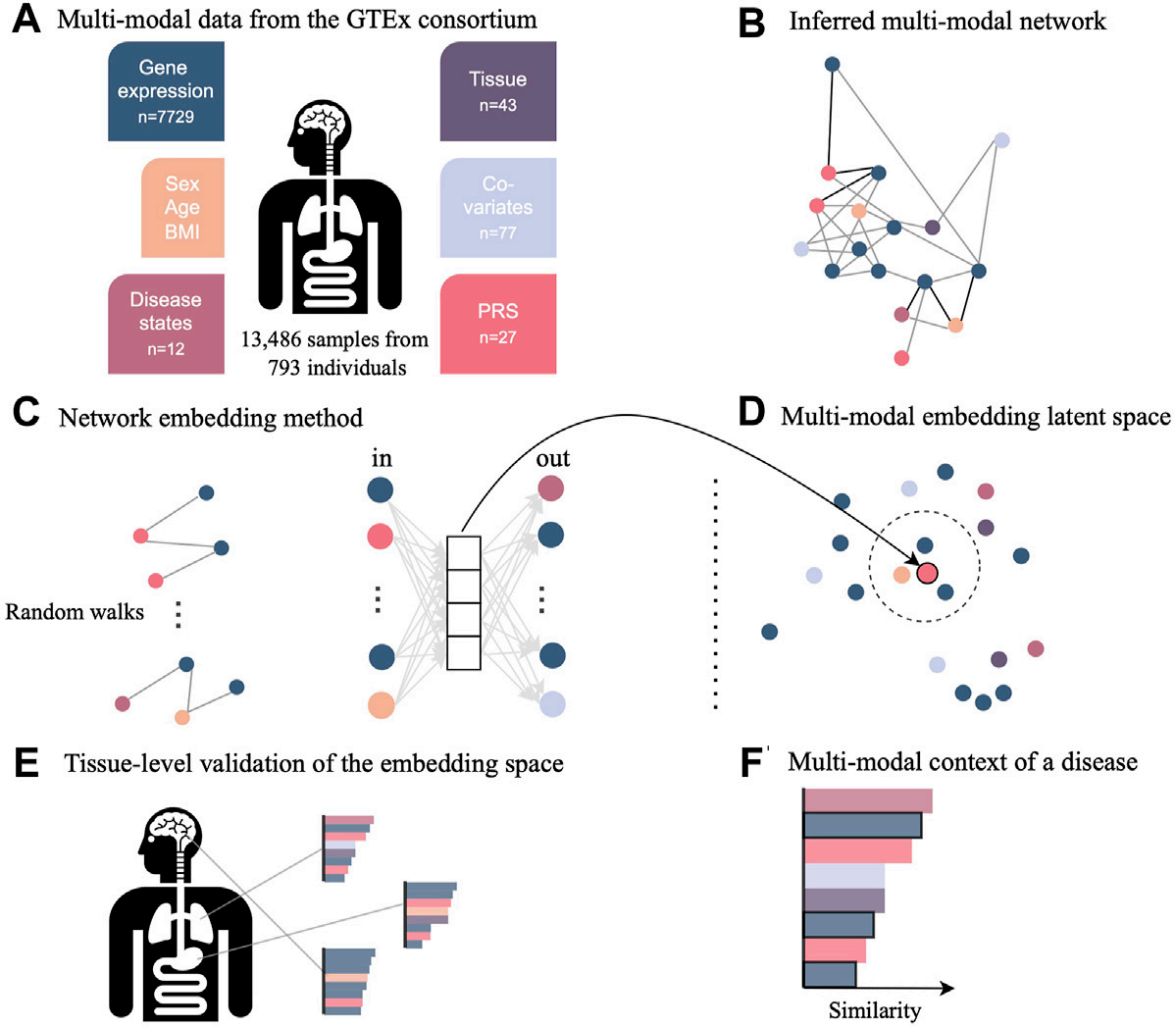
Novel network inference and embedding framework to integrate multimodal data for the investigation in low-dimensional space. **(A)** Data from the GTEx consortium consisted of gene expression across multiple tissues, phenotypes such as sex and age, diagnosis, technical and biological covariates, as well as polygenic risk scores (PRS) representing the genetic predisposition for a certain disease. **(B)** Multimodal data integration was carried out with the network inference method named KiMONo to obtain a multimodal multi-tissue network. **(C)** Resulting network was embedded into a low-dimensional space using a method adapted from GeneWalk, which uses the results of random walks of sequentially visited nodes. **(D)** Weights of the hidden layer are the embeddings and were explored using cosine similarity scores to **(E)** validate the embedding space using tissue-specific genes and **(F)** understand the multimodal context of genes essential to COVID-19, which have been identified previously by external sources.

COVID-19 disease affects multiple organs featuring symptoms from lung, neurological, hematological, liver, kidney, and heart disease. To shed light on these multimodal characteristics, we use the pre-pandemic multi-tissue GTEx cohort of close to 1,000 individuals. This cohort contains individuals with various disease diagnoses, which are referred to as comorbidities in the context of a SARS-CoV-2 infection. We established a new statistical framework to elucidate the multimodal context of any feature of interest, but especially of previously identified genes associated to COVID-19. This framework consisted of inferring a multimodal network and embedding the nodes into a low-dimensional embedding space for effective exploration of similarities between nodes and data modalities (Figure 1).

### Disease State and Polygenic Risk Capture Differential Information

Using our novel framework, we were able to integrate data of different modalities from phenotypes and gene expression of 43 tissues to the genetic risks and existing disease diagnoses. To capture the genetic risk of developing a disease, we computed polygenic risk scores (PRS) for all GTEx individuals with available genotypes by using a genome-wide scoring approach. We identified and computed PRS for 24 large GWAS of diseases and traits, which are known or suggested to bear a risk of a severe COVID-19 disease course and from three COVID-19 GWAS itself.

For an exploration of data modalities, we calculated the Pearson’s correlation between disease states, PRS, and phenotypes (Supplementary Figure S2). The correlation between PRS of diseases was high, such as for schizophrenia to bipolar, major depressive disorder (MDD), and coronary artery disease with Pearson’s correlations of 0.97, 0.62, and 0.53, respectively. Furthermore, the PRS of type II diabetes (T2D) was correlated with type I diabetes (T1D) (0.38), COVID-19 severity (0.76), stroke (0.24), and hypertension (0.46). On the other hand, the actual disease state T2D was correlated lowly with disease renal failure (0.22), hypertension (0.35), and age (0.25). However, there was almost no correlation between the genetic risk (PRS) and actual development of a disease (diagnosis as provided by GTEx). For instance, the correlation for hypertension, asthma, T1D, and T2D were 0.14, 0.09, 0.19, and 0.04, respectively. The low correlation of genetic load with disease status makes the integration of complementary information very important in the study of a disease, when the PRS covers the genetic risk to develop a certain disease, but the actual development of a disease is additionally influenced by environmental factors.

### Multimodal Network Embedding of the GTEx Cohort

**FIGURE 2:**
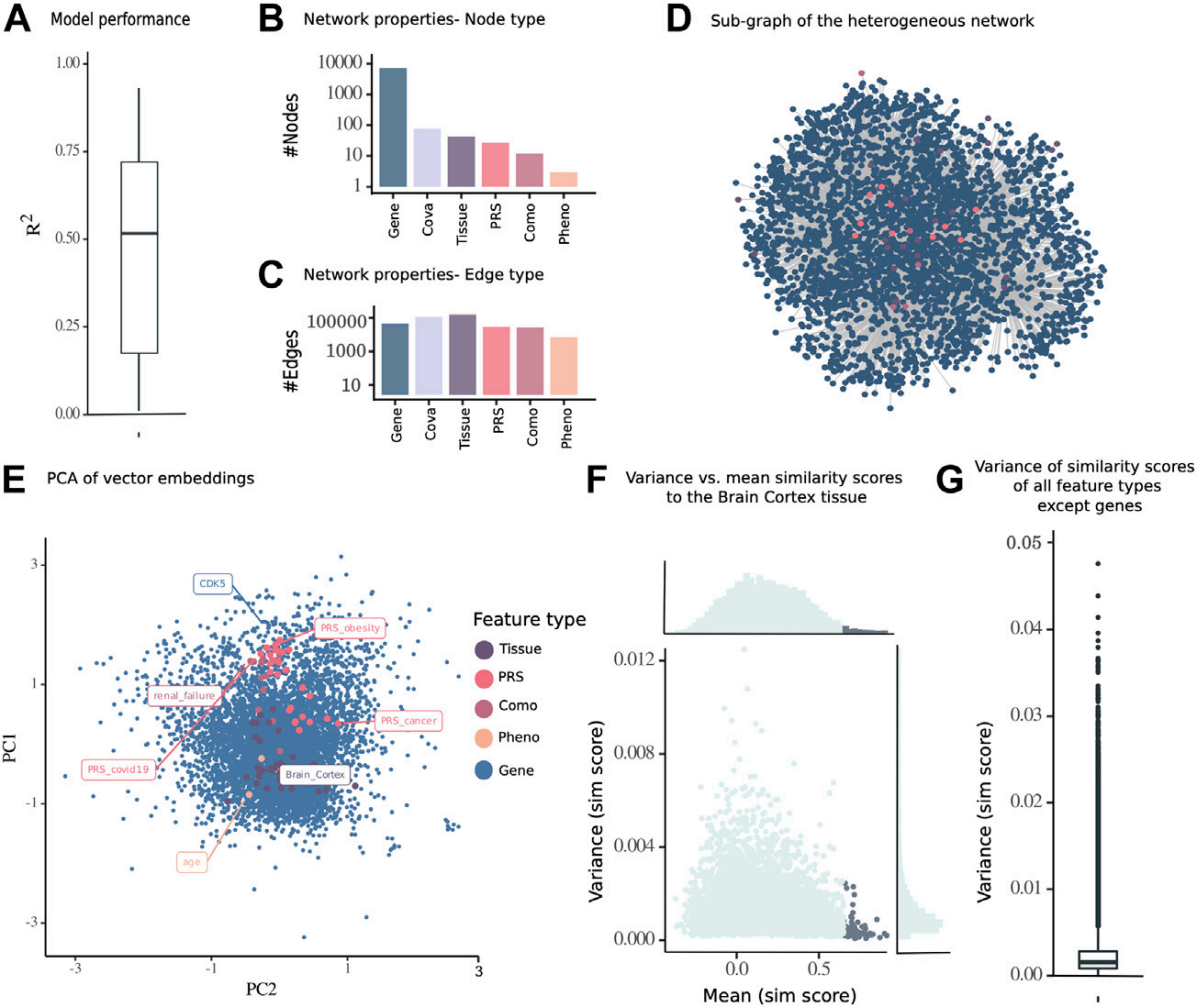
Embedding of a multimodal network consisting of nodes from different data modalities including gene expression, PRS, diseases (comorbidities of COVID-19), phenotypes, and tissues of 13,486 samples from 793 individuals from the GTEx cohort. **(A)** Subnetwork of the inferred multimodal data. **(B)** Number of nodes and **(C)** edges of the complete network. **(D)** $R^{2}$ performance of sparse group lasso gene models from KiMONo, as a quality measure of the edges of the full network. **(E)** PCA of full network embedding (1 of 100 runs) with PRS for obesity, COVID-19, and cancer, as well as CDK5 gene, brain cortex tissue, and age phenotype highlighted with labels. **(F)** For one node of interest (*brain cortex*), the highest similarity score across 100 runs was plotted against the variance across 100 runs. The nodes with the highest similarity score to the node of interest (*brain cortex* in purple) have a low standard deviation across 100 runs. Marginal density plots displayed to the sides. **(G)** Variance of similarity scores of all nongene nodes.

Multimodal GTEx data were integrated with the inference algorithm, KiMONo, and an example for one single gene model can be found in Supplementary Figure S3. Models were filtered for low performance, low beta values, and stability selection. We obtained 7,236 gene models of 7,251 with 461,216 edges. Next, after running the reverse models and retaining the statistically significant associations, the median $R^{2}$ of all the models was 0.52 (Figure 2A), and we obtained a network comprising genes (*n* = 7,202), phenotypes (*n* = 3), diseases (*n* = 12), PRS (*n* = 27), covariates (*n* = 77), and tissues (*n* = 43) (Figure 2B). While the genes were dominating the network in the number of nodes, the most common edge type was *n* = 62,902 between a gene and tissue variable, as well as *n* = 44,965 between gene and covariates, *n* = 18,521 between genes, *n* = 11,613 between the gene and PRS, *n* = 10,553 between gene and disease states, and *n* = 2,817 between the gene and phenotypes (Figure 2C).

To identify an optimal set of parameters for the embedding from the multimodal genetic risk-, gene expression-, phenotype-, and disease diagnosis-aware network, we performed a grid search on a smaller network derived from brain gene expression samples. The optimal parameters of the models were a window size = 2 of positive examples and embedding dimensionality = 16, for which the normal distributed variance (Supplementary Figure S4B) was the highest, with a mean of 0.080 across 10 repetitions (Supplementary Figure S4A). This reflected the highest information content to be captured without overfitting to the data. The network embedding algorithm was run 100 times to reduce the variability in stochastic random walks, using this set of optimal parameters for the multimodal network inference with KiMONo.

To establish a simplified visualization of the 16-dimensional embedding space, we chose one random run representative of 100 runs and subjected it to PCA and finally visualized all nodes in the first and second principal component. As the number of dimensions was optimized during the grid search process, both principal components together explained a variance of 20.17% (Figure 2D). The genes were spread across the PCA, with same structure within the other modalities. Some diseases and PRS displayed high PC1 values, such as the PRS for obesity and COVID-19 and the disease renal failure, while the PRS for cancer and many tissues had lower PC1 values (Figure 2E). While the linear PCA cannot completely represent the nonlinear embedding space, the PCA could be viewed as an approximation of the embedding space.

Furthermore, we computed the maximal similarity score across a 100 runs from the brain cortex tissue node to all other nodes and identified that the topmost similar node with a maximal similarity score above 0.65 also had a relatively low variance across 100 runs (Figure 2F). The robustness of pairwise similarity scores across 100 runs was confirmed by the low variance of similarity scores of nongenes to all other nodes (Figure 2G).

### Embedding Recapitulates the Association Between the Brain Tissue Node and Brain-Specific Genes

**FIGURE 3:**
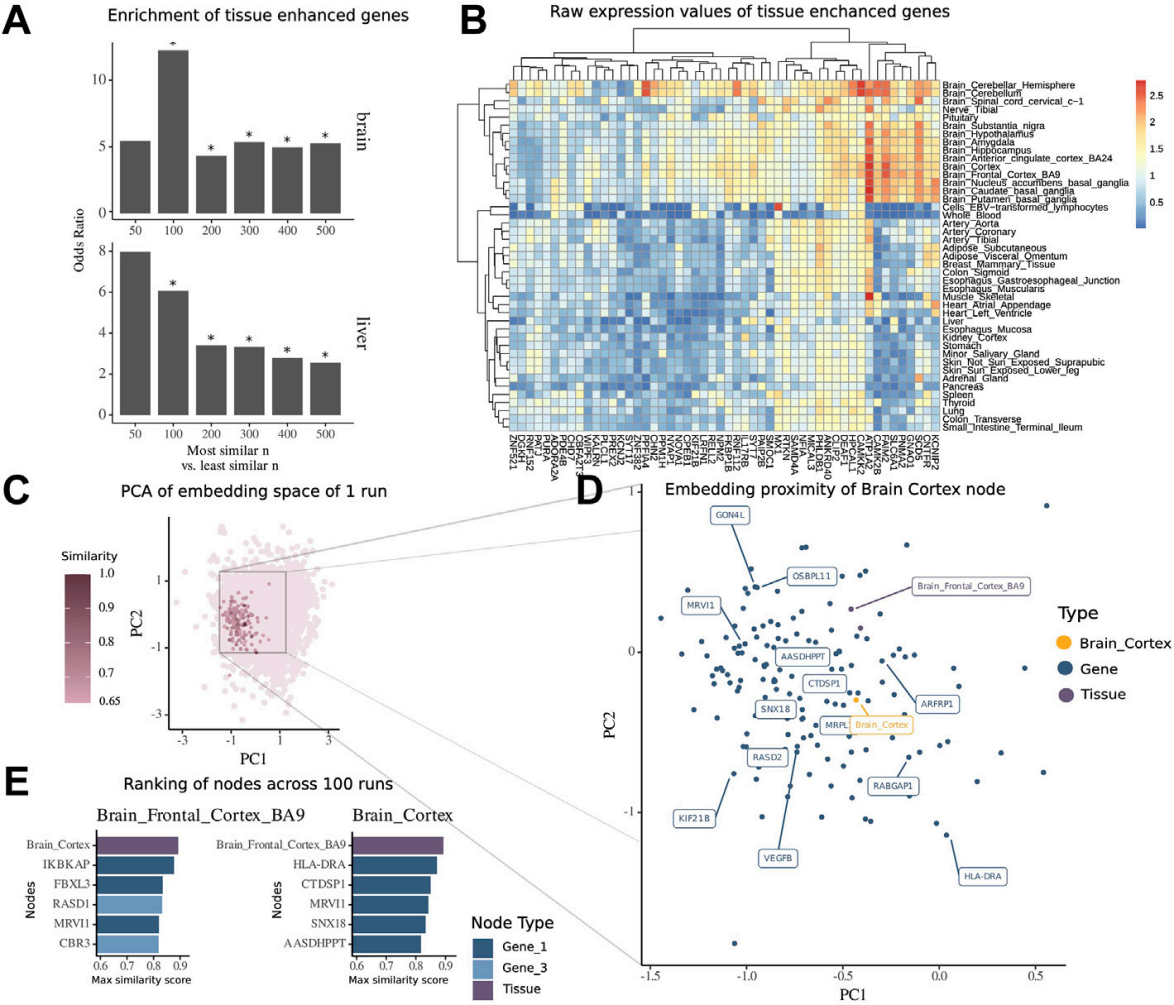
Embedding of multimodal network identifies tissue-associated genes. **(A)** Enrichment of tissue-enhanced genes within the top *n* most similar nodes compared to bottom *n* least similar nodes by mean similarity score across 100 runs of embedding in expression in the brain and liver. **(B)** Median expression per tissue within the GTEx dataset of 50 genes found to be enriched in expression in the brain among the top 500 most similar nodes. **(C)** Embedding of all nodes of the multimodal network of a representative run, highlighted are nodes with high similarity score of tissue node brain cortex. **(D)** Zoomed-in view of embedding space around brain cortex with 15 topmost similar nodes highlighted by labels. **(E)** Ranking of most similar nodes for brain cortex and brain frontal cortal BA9 across all 100 runs of embedding, with 1-hop neighbor genes in dark blue and 3-hop neighbor genes in light blue.

To validate the embedding space obtained from our multimodal network, we evaluated how much the similarity scores between gene nodes and tissue nodes recapitulated the tissue-specific expression patterns of genes. To do so, we retrieved the similarity scores from all genes with the brain tissue node and overlapped varying *n* topmost and least similar genes with genes previously reported to be enhanced in their expression in brain tissues within The Human Protein Atlas database. We found that these genes appeared more frequently in the set of most similar genes than in the set of least similar genes, thus confirming their functional role in the tissue of interest. The odds ratio of enrichment within the most similar nodes is 4.33-fold higher than that in the least similar nodes, when looking at the top and bottom *n* = 200 nodes ( $m_{top}=20$ , $m_{bottom}=5$ , $x^{2}\;test\;p\;\;value=0.0038$ ) and even 12.24-fold for *n* = 100 ( $m_{top}=11$ , $m_{bottom}=1$ , $x^{2}\;test\;p\;\;value=0.0074$ ) (Figure 3A). For further tissues which had many samples within GTEx and a high number of genes with tissue-enhanced expression within the database such as liver, the same trend was visible (Figure 3A). Finally, the enhanced tissue expression for brain-specific genes was confirmed by the expression values in transcript per million (TPM) within the GTEx dataset (Figure 3B).

For the illustration of the embedding space centered around the tissue brain cortex, we visualized the whole embedding from one single run using the principal components 1 and 2 of a PCA (Figure 3C) and then zoomed into the local proximity embedding space containing the most similar nodes (Figure 3D).

For the tissue brain cortex, one of the most similar nodes was the gene major histocompatibility complex*,* class II, DR alpha (HLA-DRA) which is shown to be elevated in expression in gliomas (Fan et al., 2017). Another node was the tissue type brain frontal cortex BA9.

To further demonstrate how the similarity scores can be used for contextualization of genes and other modalities, we extracted the contextualized top nodes for both brain cortex and brain frontal cortex across 100 embedding runs ranked by the maximum similarity score (Figure 2E). The top genes for both brain regions have been annotated in previous studies to be associated with mental disease or for the functioning of the brain. Notably, we found Ras-related dexamethasone induced 1 (RASD1), which encodes for a small GTPase overexpressed as protein in the frontal cortex (Fishilevich et al., 2016) and is associated with the pathway of nNOS signaling at neuronal synapses. Interestingly, we note that this node has a high similarity to the brain frontal cortex node (similarity score = 0.84), while it was not a direct neighbor, but a 3-hop neighbor, in the underlying multimodal network. This shows how the network embedding can capture highly relevant relationships between nodes, despite their relative distance in the underlying network.

### Embedding Elucidates Context of Known Diseases

We further illustrate the use of the embedding by exploring in more depth the relationship between nodes of various modalities and nodes of selected diseases and PRS. For instance, ischemic heart disease was found closest to hypertension, chronic obstructive pulmonary disease (COPD), and cerebrovascular disease (Supplementary Figure S4A). Among the genes, 1-hop and 2-hop neighbors in the underlying network, were chromatin licensing and DNA replication factor 1 (CDT1), minichromosome maintenance complex component 5 (MCM5), and cAMP-responsive element-binding protein 1 (CREB1), which have been linked to ischemic heart disease before. The latter has been found to be a strong genetic predictor for heart rate response by being a key player during contraction and cardiac memory (Urbanek et al., 2005; Rankinen et al., 2010; Haidar and Moni, 2020). Furthermore, within the top 15 most similar nodes, we could find the node for genetic predisposition for heart failure and the node for predisposition for COVID-19 hospitalization.

Interestingly, the disease node for major depressive disorder (MDD) was in the midst of gene nodes in the PCA with no other node modalities appearing in the 15 most similar node ranking (Supplementary Figure S5B). Most similar genes were mammalian STE20-like kinase-1 (MST1), kinase promoting apoptosis, or aryl hydrocarbon receptor nuclear translocator like 2 (ARNTL2), involved in the circadian clock regulation and found to be one of the three genome-wide associations of suicide in *MDD*. Another was cytochrome P450 family 2 subfamily E member 1 (CYP2E1), which is an important protein in the microsomal oxidation system. Shao et al. (2021) evaluated the mutual pathomechanisms in both MDD and nonalcoholic fatty liver disease as they mediate and promote the progression of each other.

As another example*, T2D was* highly similar to T1D and pneumonia, as well as the tissue tibial artery (Supplementary Figure S5C). Genes from 1- to 3-hop neighbors have been described and associated with T2D in literature before, including microtubule nucleation factor (TPX2), fibroblast growth factor receptor 1 (FGFR1), RNA helicase and ATPase (UPF1), and Huntington (HTT) (Tani et al., 2012; Montojo et al., 2017; Hall et al., 2019; Li et al., 2020).

As a final example for disease states which is also a known pathology of COVID-19, we studied pneumonia (Supplementary Figure S5D). Among the most similar nodes were acute pneumonia, T1D and T2D, as well *as tibial* artery. The increased risk of pneumonia in diagnosed patients with diabetes has been established (Vardakas et al., 2007; Ehrlich et al., 2009). Furthermore, neutrophil cytosolic factor 1 (NCF1) encodes for a component of the NADPH oxidase complex and has been associated with fibrosis, inflammation, as well as pneumonia (Zamakhchari et al., 2016). Annexin A1 (ANXA1), playing a role in innate and adaptive immune response, has also been found to control the inflammatory response. The gene has been further suggested as a prognostic biomarker for COVID-19 by decrease in severe cases (Machado et al., 2020).

Next, we investigated the most similar nodes to each of the PRS nodes, which represent the genetic risk for a certain disease. The top 15 nodes of each PRS were frequently other PRS nodes. This inflation might be due to the higher correlation of PRS among one another, as we had quantified prior to the embedding (Supplementary Figure S2). For instance, 12 of the 15 most similar nodes to the node of chronic kidney disease were genetic risk scores that ranged from coronary artery disease to COPD and Crohn’s disease (Supplementary Figure S6A), which are known comorbidities (Chen and Liao, 2016; Demir et al., 2014; Cai et al., 2013). The three other most similar nodes were genes, namely, glutathione S-transferase Mu 3 (GSTM3), scavenger receptor cysteine-rich type 1 protein M130 (CD163), and ribosomal protein S10 (RPS10) (1-, 1-, and 2-hop neighbors, respectively). All three have been described as important for the development of renal tissue, carcinomas, or as biomarkers for inflammation (Tan et al., 2013; Mejia-Vilet et al., 2020; Serin et al., 2021).

As another example, ornithine decarboxylase 1 (ODC1) (3-hop neighbor) was the topmost similar gene to the genetic risk for cancer. Kim et al. (2017) proposed ODC1 as a therapeutic target for inhibition for endometrial cancer, as it is often overexpressed and contributes to cell proliferation. Among the other similar nodes were the genetic risk for lung cancer (similarity score = 0.83) as well as for alcohol abuse (similarity score = 0.79) (Supplementary Figure S6B).

The PRS for schizophrenia was most similar to the one of *MDD* and bipolar disorder along with the PRS for COVID-19 and the one for obesity (Supplementary Figure S6C). Indeed, the connection between mental diseases and COVID-19 had been of interest, with the highest odds ratio for susceptibility and mortality in patients with severe mental disorders (Fond et al., 2021; Liu et al., 2021; Wang et al., 2021). It has been suggested that this vulnerable group exhibits lower immune function and poorer resilience. In this section, we demonstrate how the similarity score can be used for contextualization of disease states and PRS to capitulate disease-associated factors and genes.

### Embedding Uncovers Novel Dependencies Between COVID-19 and Tissues, PRS, and COVID-19 Comorbidities

Having shown the applicability of the framework to capture both tissue-specific and at the same time disease-specific genes and described associations, we aimed next at understanding host factors important for COVID-19 in its complex multimodal context of GTEx cohort data. Thus, we compiled genes from previous studies of different sources and explored their proximities in the embedding space. These were gene sets derived from GWAS, CRISPR, physical interaction studies, as well as multi-omics patient data. The top associations of these genes to tissues, PRS, and diseases (which can be considered comorbidities of COVID-19) were represented as similarity graphs that pass our threshold of similarity score >0.65. In addition, we focused on the multimodal context of genes which had a connection to at least one of the three COVID-19 genetic predispositions (PRS) of susceptibility, severity, or hospitalization.

**FIGURE 4:**
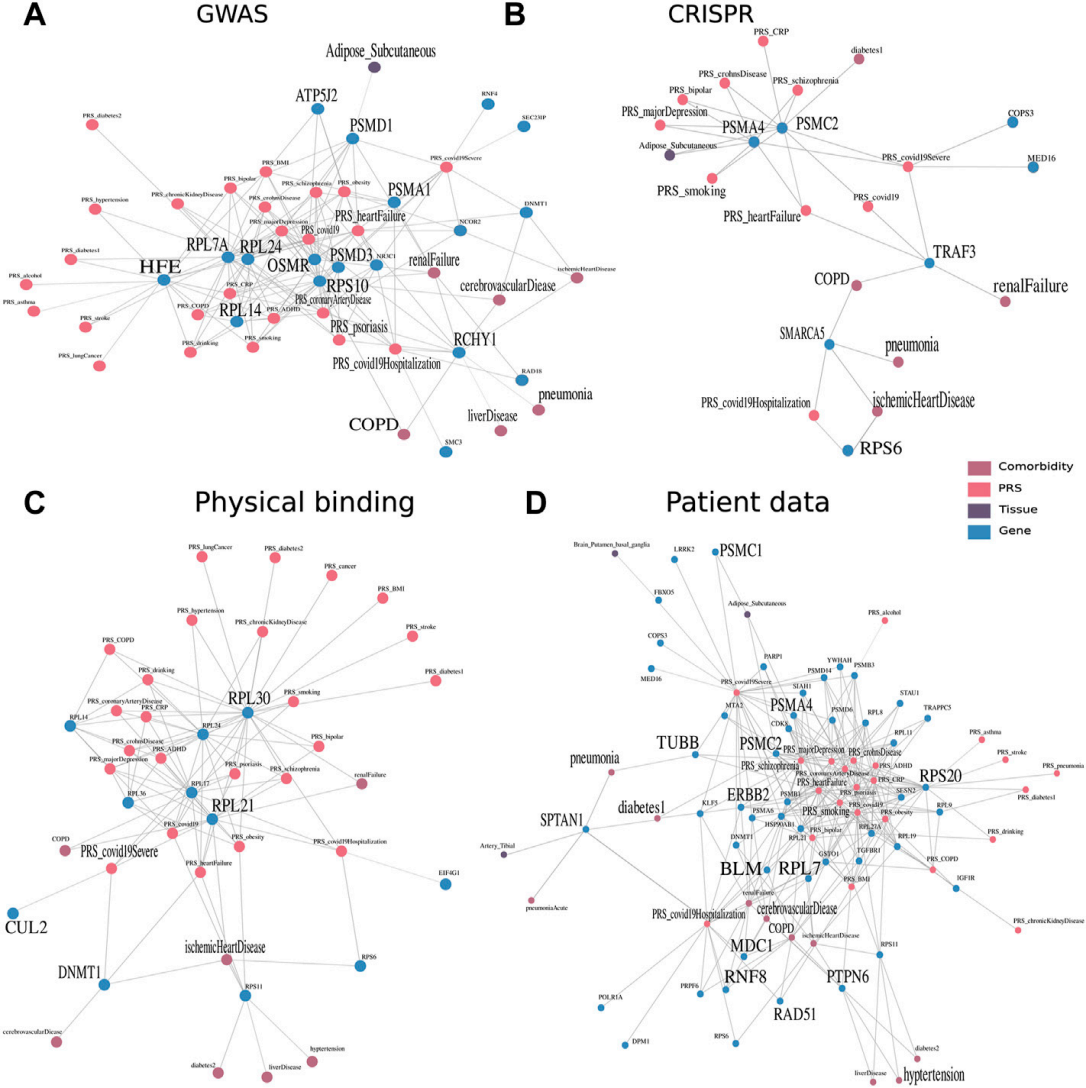
Multimodal context of known host factors important for COVID-19, stemming from different public experimental and patient data sources. Multimodal context, especially with regard to the tissues, PRS, and COVID-19 comorbidities, was represented as a network, an edge between two nodes was drawn when the similarity score within the embedding space >0.65 and robustly identified in 80 of 100 run repetitions. Similarity networks of genes derived through **(A)** GWAS studies (The COVID-19 Host Genetics Initiative 2020) and **(B)** CRISPR studies derived from Wei et al. (2021) and Schneider et al. (2021). **(C)** Furthermore, physical interaction experiments of ribonucleoprotein captures and immunoprecipitation (Gordon et al., 2020; Lee et al., 2020), as well as **(D)** patient data of whole blood samples (Shen et al., 2020; D’Alessandro et al., 2020; Di et al., 2020; Messner et al., 2020; Wu et al., 2021; Overmyer et al., 2021; Geyer et al., 2021; Demichev 2021).

First, we investigated the similarity network of genes derived from GWAS studies (Figure 4A). Small and large ribosomal subunits and factors, such as 40S ribosomal protein S10 (RPS10), 60S ribosomal protein L7a (RPL7A), 60S ribosomal protein L24 (RPL24), and 60S ribosomal protein L14 (RPL14) were connected to many genetic risks. RPS10 was additionally connected to comorbidities such as *COPD*, cerebrovascular disease, and renal failure. There is evidence of ribosomal protein entry channel blockage by the viral NSP1 during infection, thus inhibiting mRNA translation (Simeoni et al., 2021). Another notable set of proteins were factors from the proteasome, consisting of proteasome 26S subunit, non-ATPase 3 (PSMD3), proteasome 26S subunit, non-ATPase 1 (PSMD1), and proteasome 20S subunit alpha 1 (PSMA1). The latter was associated with adipose tissue and renal failure in the embedding and is involved in the maintenance of protein homeostasis involved in ATP-dependent degradation of ubiquitinated proteins, including those of coronaviruses (Tiwari et al., 2021). Ring finger and CHY zinc finger domain containing 1 (RCHY1) was associated with many comorbidities, including COPD, cerebrovascular disease, renal failure, ischemic heart disease, liver disease, and pneumonia, as well as the genetic risks of psoriasis, heart failure, and coronary artery disease. RCHY1 that was derived from the GWAS study comparing COVID-19 hospitalized versus nonhospitalized was connected to both PRS of COVID-19 and COVID*-*19 hospitalization. This protein is also involved in E3-dependent ubiquitination and proteasomal degradation, including tumor protein 53 (TP53), histone deacetylase 1 (HDAC1), and cyclin-dependent kinase inhibitor 1B (CDKN1B), thus regulating their levels and cell cycle progression. While ribosomes and proteases were connected to many genetic risks, RCHY1 was further similar to many developed diseases that were reported to be comorbidities of COVID-19.

Proteasome 26S subunit, ATPase 2 (PSMC2) and proteasome 20S subunit Alpha 4 (PSMA4) identified by a CRISPR study (Schneider et al., 2021) were associated with many genetic risks of diseases, ranging from *heart* failure to MDD (Figure 4B). The tumor necrosis factor receptor associated factor 3 (TRAF3) identified by Wei et al. (2021) study was on the other hand associated with the diseases renal failure and COPD and has been annotated by previous studies in signal transduction for activation of immune and antiviral responses. The risk of hospitalization with COVID-19 was associated with the genes small ribosomal protein S6 (RPS6) and a member of the SWI/SNF family involved in ATP-dependent chromatin remodeling complexes (SMARCA5) identified by Schneider et al. (2021) and RuthHanna et al. (2021), respectively. They were further connected to comorbidities of pneumonia and ischemic heart disease occurring in the individuals. Gupta and Nayak (2021) focused on SMARCA4, which is a paralog, acting as the catalytic subunit of the SWI/SNF remodeling complex, regulating chromatin structure.

Viral factors were found to physically interact with large ribosomal subunits from the 40S and 60S protein. Ribosomal protein L30 (RPL30) and ribosomal protein L21 (RPL21) identified by Lee et al. (2020) were well-connected to many different diseases (Figure 4C). While RPL30 was connected to almost all genetic risks including all three COVID-19 genetic risk nodes, RPL21 was additionally connected to the comorbidity of ischemic heart disease which is common in patients hospitalized with COVID-19. DNA (cytosine-5)-methyltransferase 1 (DNMT1) and Cullin (CUL2) identified by Gordon et al. (2020) were found to have interesting functional annotations of DNA methylation maintaining methylation patterns and ubiquitination for the marking of proteins for degradation.

Finally, accumulated over the eight serum or plasma-derived proteome and transcriptome patient data studies, we detected close similarity to genes and proteins of four studies. Eight genes encode for subunits of proteasomes (Figure 4D). They were connected most frequently to MDD and schizophrenia, as well as Crohn’s disease, heart failure, and smoking. Three of the eight factors, namely, PSMC1, PSMC2, and PSMA4 were in addition associated with adipose tissue and the risk of a severe case of COVID-19. The 11 subunits of ribosomes (seven RPL, three RPS, and one RNF) were connected to a variety of PRS, predominantly, heart failure, MDD, and obesity.

Protein tyrosine phosphatase non-receptor type 6 (PTPN6) was associated with one COVID-19 node and with the highest number of diseases (7) from COPD over hypertension to cerebrovascular disease. This protein tyrosine phosphatase regulates a variety of cellular processes from cell growth to oncogenic transformation. It has also been shown to be expressed in B cells in severe COVID-19 (Stephenson et al., 2021). In addition, tubulin beta class I (TUBB) and spectrin alpha, non-erythrocytic 1 (SPTAN1) are both involved in the cytoskeleton, encoding for beta tubulin and scaffolding proteins, respectively. The multi-omic context of SPTAN1 proved to be interesting by being connected to pneumonia and T2D as comorbidity and tibial artery as tissue.

Some genes that were identified over the serum and plasma patient data are involved in the cellular response to DNA damage and repair, namely, RAD51 recombinase (RAD51), mediator of DNA damage checkpoint 1 (MDC1), bloom syndrome RecQ-like helicase (BLM), and Erb-B2 receptor tyrosine kinase 2 (ERBB2). ERBB2, belonging to the epidermal growth factor receptor family of receptor tyrosine kinases, had also been previously linked to the cytokine release storm and thus severity of an infection with SARS-CoV-2 (Khitan et al., 2022). This gene was especially of interest in combination with obesity and gut microbiome in COVID-19 patients (Khitan et al., 2022). Finally, we explored the tissue context of COVID-19–associated genes, identified in blood of mostly severe patients, independently if they were additionally associated to the genetic predisposition. By counting the number of tissues in close proximity, we found that brain-related tissues such as nucleus accumbens basal ganglia, substantia nigra, or spinal cord were among the most frequently discovered, along with tibial artery, small intestine, and pituitary (Table 1). During the acute phase of infection, SARS-CoV-2 was found in the substantia nigra, where it preferentially targeted dopaminergic neurons (Bouali‐Benazzouz and Benazzouz, 2021). It has been discussed that the infection of brain tissue could trigger cellular processes linked to neurodegeneration, and its course has been shown to worsen diseases such as Parkinsonism (Bouali‐Benazzouz and Benazzouz, 2021). Lehmann et al. (2021) described that the small intestine was affected in COVID-19 patients. Frara et al. (2021) showed poorer outcomes for patients with pituitary dysfunction, affecting patients with an abnormal endocrine phenotype such as hypopituitarism and diabetes. Also, COVID-19 patients exhibit worse outcomes with thrombosis in the tibial arteries (Singh et al., 2021).

**TABLE 1:** Top five tissue context in embedding of known COVID-19 genes discovered by different study types, expressed as counts and proportion of all tissues.

	Tissue	Count	Proportion of all tissues
GWAS	Brain spinal cord cervical c-1	27	0.046
	Breast mammary tissue	22	0.0375
	Brain frontal cortex BA9	22	0.0375
	Cells EBV-transformed lymphocytes	22	0.0375
	Heart atrial appendage	22	0.0375
CRISPR	Brain nucleus accumbens basal ganglia	4	0.0656
	Artery tibial	4	0.0656
	Adipose subcutaneous	4	0.0656
	Brain anterior cingulate cortex BA24	3	0.0492
	Breast mammary tissue	3	0.0492
Physical interaction	Brain substantia nigra	9	0.0811
	Pituitary	8	0.0721
	Small intestine terminal ileum	5	0.045
	Pancreas	5	0.045
	Adipose visceral omentum	5	0.045
Patient	Brain spinal cord cervical c-1	142	0.0433
	Breast mammary tissue	127	0.0388
	Stomach	126	0.0385
	Pituitary	122	0.0372
	Cells EBV-transformed lymphocytes	118	0.036

## Discussion

We established a two-step framework that allows us to integrate multi-omic data across multiple tissues and given genetic risk and disease state information in order to better understand diseases, especially COVID-19, in their complex context. First, a multimodal network was inferred using KiMONo which associates genes with all other data modalities, consisting of genes, tissues, phenotypes, comorbidities, and PRS, using multiple regression. Second, all nodes were projected into an embedding space using the method GeneWalk. For efficient analysis of the embedding, similarity scores were employed, under the assumption that associated nodes are in the proximity within the embedding space. We showed that the multimodal network from the GTEx pre-corona population cohort was of high quality being able to achieve high gene model performances using the $R^{2}$ measures. Furthermore, we assured the quality of the embeddings by optimizing the parameters such that we maximize the variance of the resulting node pair cosine similarity distribution. The fact that similarity scores between any two vectors had low standard deviation across the 100 runs reflects the robustness of the resulting embeddings.

We validated the relevance of the network embedding by showing that tissue-specific genes were significantly more enriched in the set of genes with the highest similarity scores to the tissue of interest, as compared with the set of genes with the lowest similarity scores. Thus, our method was able to capture the most important factors for each node of interest. The embedding was also validated by recapitulating disease-related factors such as for the disease ischemic heart disease, T2D, and MDD or the genetic predisposition for chronic kidney disease and cancer. Interestingly, those top similar nodes come from a diverse degree of neighborhood of the initial KiMONo network, which confirms that the embedding helps capture highly relevant relationships between nodes. In summary, we were able to (1) prioritize important factors from multitude of connections in the original network and (2) pick up factors that were connected over 1, 2, and 3 hops. With the embedding, the relationship between any two nodes could be examined in the full network context and does not need to be restricted to directly connected ones. The information gain exceeding the original network is due to the embedding which is learned by using sequences within random walks as training examples. In this way, the larger context of each node is being taken into account for the embedding.

We delineated the multimodal context of previously identified COVID-19 genes. Strikingly, different experimental types captured different information. While the networks from genes that were derived from physical interaction experiments were able to elucidate the roles of ribosomal proteins, the genes from CRISPR studies had a focus on proteases and genes that had COVID-19 comorbidities in their proximity such as SMARCA5 and TRAF3, involved in chromatin remodeling and activation of viral response. Furthermore, the networks from whole blood samples of patients revealed the role of many proteases and ribosomes that had many genetic risks in their proximity. Others were coupled to functions such as DNA repair, ubiquitination, and functions within the cytoskeleton. Another interesting gene only present in the patient’s blood data was PTPN6; it was associated with the highest amount of comorbidities, ranging from COPD to hypertension. This gene was less associated with the genetic risk for different diseases but with the actual development of them, making this gene a central player for COVID-19 when other comorbidities were already present. Importantly, we also delineate that in this disease, many tissues were involved, including the brain and small intestine.

The framework’s limitations are the prior used to establish gene–gene links. Our analysis was limited to around 7,000 genes mainly due to lack of mappability to the prior, but could be extended to the full set of human protein coding genes. In addition, we limited the PRS only to GWAS studies, selecting the diseases that have been found to be most relevant for COVID-19, while many others are of potential relevance in a cross-tissue cross-disease cohort. Our network could also be used to understand the context of other diseases by expanding the PRS or diseases being integrated into the network.

The power of our analysis stems from the large cohort, enabling the contextualization of the modality impact of a disease on a population level and across different tissues. The considerable amount of samples gives it statistical power, especially in the brain tissues. To our knowledge, this is the first time that the genetic predisposition to COVID-19 has been analyzed using a pre-corona population cohort spanning multiple tissues in the body, while taking the genetic setting, the developed diseases, and phenotypes into account. This allowed for the understanding of this complex disease on many layers from genetics to comorbidities influenced by environmental factors, especially when the information they covered was complementary.

## Data Availability

The data analyzed in this study are subject to the following licenses/restrictions: The gene expression data and covariates v8 used for the analyses described in this study were obtained from the publicly available GTEx Portal on 10/08/19 and complemented by genomic and phenotypic information which are not readily available because of data privacy reasons. Requests to access the datasets should be directed to the dbGAP portal. We retrieved genomic and phenotypic information from dbGaP accession number phs000424.GTEx.v8.p2.c1 on 06/08/2021. Requests to access these datasets should be directed to https://www.ncbi.nlm.nih.gov/projects/gap/cgi-bin/study.cgi?study_id=phs000424.v8.p2.

## References

[B1] Argelaguet R. Velten B. Arnol D. Dietrich S. Zenz T. Marioni J. C. (2018). ‘Multi-Omics Factor Analysis—A Framework for Unsupervised Integration of Multi-Omics Data Sets’. Mol. Syst. Biol. 14, e8124. 10.15252/msb.20178124 PubMed Abstract | 10.15252/msb.20178124 | Google Scholar 29925568 PMC6010767

[B2] Bouali-Benazzouz R. Benazzouz A. (2021). ‘Covid-19 Infection and Parkinsonism: Is There a Link?’, Mov. Disord. 36 (8), 1737–1743. 10.1002/mds.28680 PubMed Abstract | 10.1002/mds.28680 | Google Scholar 34080714 PMC8242862

[B3] Cai Q. Mukku V. K. Ahmad M. (2013). Coronary Artery Disease in Patients with Chronic Kidney Disease: a Clinical Update. Curr. Cardiol. Rev. 9 (4), 331–339. 10.2174/1573403X10666140214122234 PubMed Abstract | 10.2174/1573403X10666140214122234 | Google Scholar 24527682 PMC3941098

[B4] Carithers L. J. Ardlie K. Barcus M. Branton P. A. Britton A. Buia S. A. (2015). A Novel Approach to High-Quality Postmortem Tissue Procurement: The GTEx Project. Biopreservation Biobanking 13 (5), 311–319. 10.1089/bio.2015.0032 PubMed Abstract | 10.1089/bio.2015.0032 | Google Scholar 26484571 PMC4675181

[B5] Chang C. C. Chow C. C. Tellier L. C. Vattikuti S. Purcell S. M. Lee J. J. (2015). Second-generation PLINK: Rising to the Challenge of Larger and Richer Datasets. GigaSci 4 (1), 7. 10.1186/s13742-015-0047-8 PubMed Abstract | 10.1186/s13742-015-0047-8 | Google Scholar PMC4342193 25722852

[B6] Chen C. Y. Liao K. M. (2016). Chronic Obstructive Pulmonary Disease Is Associated with Risk of Chronic Kidney Disease: A Nationwide Case-Cohort Study. Sci. Rep. 6, 25855. 10.1038/srep25855 PubMed Abstract | 10.1038/srep25855 | Google Scholar 27166152 PMC4863146

[B7] D’Alessandro A. Thomas T. Dzieciatkowska M. Hill R. C. Francis R. O. Hudson K. E. (2020). ‘Serum Proteomics in COVID-19 Patients: Altered Coagulation and Complement Status as a Function of IL-6 Level’. J. Proteome Res. 19 (11), 4417–4427. 10.1021/acs.jproteome.0c00365 PubMed Abstract | 10.1021/acs.jproteome.0c00365 | Google Scholar 32786691 PMC7640953

[B8] Demichev V. (2021). ‘A Time-Resolved Proteomic and Prognostic Map of COVID-19’. Cell Syst. 12, 780–794. e7. 10.1016/j.cels.2021.05.005 PubMed Abstract | 10.1016/j.cels.2021.05.005 | Google Scholar 34139154 PMC8201874

[B9] Demir M. E. Ercan Z. Karakas E. Y. Ulas T. Buyukhatipoglu H. (2014). Crohnic Kidney Disease: Recurrent Acute Kidney Failure in a Patient with Crohn's Disease. N. Am. J. Med. Sci. 6 (12), 648–649. 10.4103/1947-2714.147983 PubMed Abstract | 10.4103/1947-2714.147983 | Google Scholar 25599054 PMC4290055

[B10] Di B. Jia H. Luo O. J. Lin F. Li K. Zhang Y. (2020). Identification and Validation of Predictive Factors for Progression to Severe COVID-19 Pneumonia by Proteomics. Signal Transduct. Target Ther. 5, 217. 10.1038/s41392-020-00333-1 PubMed Abstract | 10.1038/s41392-020-00333-1 | Google Scholar 33011738 PMC7532335

[B11] Ehrlich S. F. Quesenberry C. P. Jr. Van Den Eeden S. K. Shan J. Ferrara A. (2009). Patients Diagnosed With Diabetes Are at Increased Risk for Asthma, Chronic Obstructive Pulmonary Disease, Pulmonary Fibrosis, and Pneumonia but Not Lung Cancer. Diabetes Care 33 (1), 55–60. 10.2337/dc09-0880 PubMed Abstract | 10.2337/dc09-0880 | Google Scholar 19808918 PMC2797986

[B12] Elezkurtaj S. Greuel S. Ihlow J. Michaelis E. G. Bischoff P. Kunze C. A. (2021). Causes of Death and Comorbidities in Hospitalized Patients with COVID-19. Sci. Rep. 11 (1), 4263. 10.1038/s41598-021-82862-5 PubMed Abstract | 10.1038/s41598-021-82862-5 | Google Scholar 33608563 PMC7895917

[B13] Ellinghaus D. Degenhardt F. Bujanda L. Buti M. Albillos A. Invernizzi P. (2020). ‘Genomewide Association Study of Severe Covid-19 with Respiratory Failure’. N. Engl. J. Med. 383, 1522–1534. 10.1056/NEJMoa2020283 PubMed Abstract | 10.1056/NEJMoa2020283 | Google Scholar 32558485 PMC7315890

[B14] Fan X. Liang J. Wu Z. Shan X. Qiao H. Jiang T. (2017). Expression of HLA-DR Genes in Gliomas: Correlation with Clinicopathological Features and Prognosis. Chin. Neurosurg. Jl 3 (1), 27. 10.1186/s41016-017-0090-7 10.1186/s41016-017-0090-7 | Google Scholar

[B15] Fishilevich S. Zimmerman S. Kohn A. Iny Stein T. Olender T. Kolker E. (2016). ‘Genic Insights from Integrated Human Proteomics in GeneCards’. Database J. Biol. Databases Curation 2016, baw030. 10.1093/database/baw030 PubMed Abstract | 10.1093/database/baw030 | Google Scholar PMC4820835 27048349

[B16] Fond G. Nemani K. Etchecopar-Etchart D. Loundou A. Goff D. C. Lee S. W. (2021). Association Between Mental Health Disorders and Mortality Among Patients With COVID-19 in 7 Countries. JAMA Psychiatry 78 (11), 1208–1217. 10.1001/jamapsychiatry.2021.2274 PubMed Abstract | 10.1001/jamapsychiatry.2021.2274 | Google Scholar 34313711 PMC8317055

[B17] Frara S. Allora A. Castellino L. Filippo L. Loli P. Giustina A. (2021). ‘COVID-19 and the Pituitary’. Pituitary 24, 465–481. 10.1007/s11102-021-01148-1 PubMed Abstract | 10.1007/s11102-021-01148-1 | Google Scholar 33939057 PMC8089131

[B18] Ge T. Chen C.-Y. Ni Y. Feng Y.-C. A. Smoller J. W. (2019). Polygenic Prediction via Bayesian Regression and Continuous Shrinkage Priors. Nat. Commun. 10 (1), 1776. 10.1038/s41467-019-09718-5 PubMed Abstract | 10.1038/s41467-019-09718-5 | Google Scholar 30992449 PMC6467998

[B19] Ge Tian. (2018). PRS-CS. Python 2022. Available at: https://github.com/getian107/PRScs . Google Scholar

[B20] Geyer Philipp. E. Arend Florian. M. Doll Sophia. Louiset Marie-Luise. Virreira Sebastian. Mu Johannes. B. (2021). ‘High-resolution Serum Proteome Trajectories in COVID-19 Reveal Patient-specific Seroconversion’. EMBO Mol. Med. 13, e14167. 10.15252/emmm.202114167 PubMed Abstract | 10.15252/emmm.202114167 | Google Scholar 34232570 PMC8687121

[B21] Gordon D. E. Hiatt J. Bouhaddou M. Rezelj V. V. Ulferts S. Braberg H. (2020). ‘Comparative Host-Coronavirus Protein Interaction Networks Reveal Pan-Viral Disease Mechanisms’. Science 370 (6521), eabe9403. 10.1126/science.abe9403 PubMed Abstract | 10.1126/science.abe9403 | Google Scholar 33060197 PMC7808408

[B22] Gupta A. Madhavan M. V. Sehgal K. Nair N. Mahajan S. Sehrawat T. S. (2020). Extrapulmonary Manifestations of COVID-19. Nat. Med. 26 (7), 1017–1032. 10.1038/s41591-020-0968-3 PubMed Abstract | 10.1038/s41591-020-0968-3 | Google Scholar 32651579 PMC11972613

[B23] Haidar M. N. Islam M. B. Chowdhury U. N. Rahman M. R. Huq F. Quinn J. M. W. (2020). Network‐based Computational Approach to Identify Genetic Links between Cardiomyopathy and its Risk Factors. IET Syst. Biol. 14 (2), 75–84. 10.1049/iet-syb.2019.0074 PubMed Abstract | 10.1049/iet-syb.2019.0074 | Google Scholar 32196466 PMC8687405

[B24] Hall E. Jönsson J. Ofori J. K. Volkov P. Perfilyev A. Dekker Nitert M. (2019). Glucolipotoxicity Alters Insulin Secretion via Epigenetic Changes in Human Islets. Diabetes 68 (10), 1965–1974. 10.2337/db18-0900 PubMed Abstract | 10.2337/db18-0900 | Google Scholar 31420409

[B25] Ietswaart R. Gyori B. M. Bachman J. A. Sorger P. K. Churchman L. S. (2021). GeneWalk Identifies Relevant Gene Functions for a Biological Context Using Network Representation Learning. Genome Biol. 22 (1), 55. 10.1186/s13059-021-02264-8 PubMed Abstract | 10.1186/s13059-021-02264-8 | Google Scholar 33526072 PMC7852222

[B26] Khitan Z. J. Chin K. V. Sodhi K. Kheetan M. Alsanani A. Shapiro J. I. (2022). ‘Gut Microbiome and Diet in Populations with Obesity: Role of the Na+/K+-ATPase Transporter Signaling in Severe COVID-19’. Obes. (Silver Spring) 30, 869–873. 10.1002/oby.23387 10.1002/oby.23387 | Google Scholar PMC8957587 35048549

[B27] Kim H. I. Schultz C. R. Buras A. L. Friedman E. Fedorko A. Seamon L. (2017). ‘Ornithine Decarboxylase as a Therapeutic Target for Endometrial Cancer’. PLOS ONE 12 (12), e0189044. 10.1371/journal.pone.0189044 PubMed Abstract | 10.1371/journal.pone.0189044 | Google Scholar 29240775 PMC5730160

[B28] Lee S. Lee Y.-S. Choi Y. Son A. Park Y. Lee K.-M. (2020). The SARS-CoV-2 RNA Interactome. Mol. Cell. 81, 2838–2850. e6. 10.1016/j.molcel.2021.04.022 10.1016/j.molcel.2021.04.022 | Google Scholar PMC8075806 33989516

[B29] Lehmann M. Allers K. Heldt C. Meinhardt J. Schmidt F. Rodriguez-Sillke Y. (2021). Human Small Intestinal Infection by SARS-CoV-2 Is Characterized by a Mucosal Infiltration with Activated CD8+ T Cells. Mucosal Immunol. 14 (6), 1381–1392. 10.1038/s41385-021-00437-z PubMed Abstract | 10.1038/s41385-021-00437-z | Google Scholar 34420043 PMC8379580

[B30] Li M. M. Huang K. Zitnik M. (2021). Graph Representation Learning in Biomedicine. Soc. Inf. Netw [Preprint]. Available at: https://arxiv.org/pdf/2104.04883.pdf . Google Scholar 10.1038/s41551-022-00942-x PMC10699434 36316368

[B31] Li J. Liu H. Srivastava S. P. Hu Q. Gao R. Li S. (2020). Endothelial FGFR1 (Fibroblast Growth Factor Receptor 1) Deficiency Contributes Differential Fibrogenic Effects in Kidney and Heart of Diabetic Mice. Hypertension 76 (6), 1935–1944. 10.1161/HYPERTENSIONAHA.120.15587 PubMed Abstract | 10.1161/HYPERTENSIONAHA.120.15587 | Google Scholar 33131311

[B32] Liu L. Ni S.-Y. Yan W. Lu Q.-D. Zhao Y.-M. Xu Y.-Y. (2021). Mental and Neurological Disorders and Risk of COVID-19 Susceptibility, Illness Severity and Mortality: A Systematic Review, Meta-Analysis and Call for Action. EClinicalMedicine 40, 101111. 10.1016/j.eclinm.2021.101111 PubMed Abstract | 10.1016/j.eclinm.2021.101111 | Google Scholar 34514362 PMC8424080

[B33] Machado M. G. Tavares L. P. Souza G. V. S. Queiroz‐Junior C. M. Ascenção F. R. Lopes M. E. (2020). The Annexin A1/FPR2 Pathway Controls the Inflammatory Response and Bacterial Dissemination in Experimental Pneumococcal Pneumonia. FASEB J. 34 (2), 2749–2764. 10.1096/fj.201902172R PubMed Abstract | 10.1096/fj.201902172R | Google Scholar 31908042

[B34] Mejia-Vilet J. M. Zhang X. L. Cruz C. Cano-Verduzco M. L. Shapiro J. P. Nagaraja H. N. (2020). Urinary Soluble CD163: a Novel Noninvasive Biomarker of Activity for Lupus Nephritis. J. Am. Soc. Nephrol. 31 (6), 1335–1347. 10.1681/ASN.2019121285 PubMed Abstract | 10.1681/ASN.2019121285 | Google Scholar 32300067 PMC7269356

[B35] Melé M. Ferreira P. G. Reverter F. DeLuca D. S. Monlong J. Sammeth M. (2015). The Human Transcriptome across Tissues and Individuals. Science 348 (6235), 660–665. 10.1126/science.aaa0355 PubMed Abstract | 10.1126/science.aaa0355 | Google Scholar 25954002 PMC4547472

[B36] Messner C. B. Demichev V. Wendisch D. Michalick L. White M. Freiwald A. (2020). Ultra-High-Throughput Clinical Proteomics Reveals Classifiers of COVID-19 Infection. Cell Syst. 11 (1), 11–24. e4. 10.1016/j.cels.2020.05.012 PubMed Abstract | 10.1016/j.cels.2020.05.012 | Google Scholar 32619549 PMC7264033

[B37] Mikolov T. Chen K. Corrado G. Dean J. (2013). “Efficient Estimation of Word Representations in Vector Space,” in 1st International Conference on Learning Representations, ICLR 2013, Scottsdale, Arizona, USA, May 2-4, 2013. Google Scholar

[B38] Mikolov T. Sutskever I. Chen K. Corrado G. Dean J. (2013). “Distributed Representations of Words and Phrases and Their Compositionality,” in Proceedings of the 26th International Conference on Neural Information Processing Systems, Air Canada, 05 December 2013, 2, 3111–3119. 10.48550/arXiv.1310.4546 10.48550/arXiv.1310.4546 | Google Scholar

[B39] Montaldo C. Messina F. Abbate I. Antonioli M. Bordoni V. Aiello A. (2021). Multi-omics Approach to COVID-19: a Domain-Based Literature Review. J. Transl. Med. 19 (1), 501. 10.1186/s12967-021-03168-8 PubMed Abstract | 10.1186/s12967-021-03168-8 | Google Scholar 34876157 PMC8649311

[B40] Montojo M. T. Aganzo M. González N. (2017). Huntington's Disease and Diabetes: Chronological Sequence of its Association. Jhd 6 (3), 179–188. 10.3233/JHD-170253 PubMed Abstract | 10.3233/JHD-170253 | Google Scholar 28968242 PMC5676851

[B41] Nelson W. Zitnik M. Wang B. Leskovec J. Goldenberg A. Sharan R. (2019). To Embed or Not: Network Embedding as a Paradigm in Computational Biology. Front. Genet. 10, 381. 10.3389/fgene.2019.00381 PubMed Abstract | 10.3389/fgene.2019.00381 | Google Scholar 31118945 PMC6504708

[B42] Ogris C. Hu Y. Arloth J. Müller N. S. (2021). Versatile Knowledge Guided Network Inference Method for Prioritizing Key Regulatory Factors in Multi-Omics Data. Sci. Rep. 11 (1), 6806. 10.1038/s41598-021-85544-4 PubMed Abstract | 10.1038/s41598-021-85544-4 | Google Scholar 33762588 PMC7990936

[B43] Oughtred R. Stark C. Breitkreutz B. J. Rust J. Boucher L. Chang C. (2019). The BioGRID Interaction Database: 2019 Update. Nucleic Acids Res. 47 (D1), D529–D541. 10.1093/nar/gky1079 PubMed Abstract | 10.1093/nar/gky1079 | Google Scholar 30476227 PMC6324058

[B44] Overmyer K. A. Shishkova E. Miller I. J. Balnis J. BernsteinBernstein M. N. Peters-ClarkePeters-Clarke T. M. (2021). Large-Scale Multi-Omic Analysis of COVID-19 Severity. Cell Syst. 12 (1), 23–40. e7. 10.1016/j.cels.2020.10.003 PubMed Abstract | 10.1016/j.cels.2020.10.003 | Google Scholar 33096026 PMC7543711

[B45] Perozzi B. Al-Rfou R. Skiena S. (2014). “DeepWalk: Online Learning of Social Representations” [Preprint]. Available at: 10.48550/arXiv.1403.6652 . Google Scholar

[B46] Rankinen T. Argyropoulos G. Rice T. Rao D. C. Bouchard C. (2010). CREB1 Is a Strong Genetic Predictor of the Variation in Exercise Heart Rate Response to Regular Exercise. Circ. Cardiovasc Genet. 3 (3), 294–299. 10.1161/CIRCGENETICS.109.925644 PubMed Abstract | 10.1161/CIRCGENETICS.109.925644 | Google Scholar 20407090 PMC3045864

[B47] Ritchie S. (2014). LiftOverPlink. Python 2022. Available at: https://github.com/sritchie73/liftOverPlink . Google Scholar

[B48] Saha A. Kim Y. Gewirtz A. D. H. Jo B. Gao C. McDowell I. C. (2017). Co-expression Networks Reveal the Tissue-specific Regulation of Transcription and Splicing. Genome Res. 27 (11), 1843–1858. 10.1101/gr.216721.116 PubMed Abstract | 10.1101/gr.216721.116 | Google Scholar 29021288 PMC5668942

[B49] Schneider W. M. Luna J. M. Hoffmann H.-H. Sánchez-Rivera F. J. Leal A. A. Ashbrook A. W. (2021). Genome-Scale Identification of SARS-CoV-2 and Pan-Coronavirus Host Factor Networks. Cell 184 (1), 120–132. e14. 10.1016/j.cell.2020.12.006 PubMed Abstract | 10.1016/j.cell.2020.12.006 | Google Scholar 33382968 PMC7796900

[B50] Serin N. Dihazi G. H. Tayyeb A. Lenz C. Müller G. A. Zeisberg M. (2021). Calreticulin Deficiency Disturbs Ribosome Biogenesis and Results in Retardation in Embryonic Kidney Development. Ijms 22 (11), 5858. 10.3390/ijms22115858 PubMed Abstract | 10.3390/ijms22115858 | Google Scholar 34070742 PMC8198291

[B51] Shao Q. Wu Y. Ji J. Xu T. Yu Q. Ma C. (2021). Interaction Mechanisms Between Major Depressive Disorder and Non-alcoholic Fatty Liver Disease. Front. Psychiatry 12, 711835. 10.3389/fpsyt.2021.711835 PubMed Abstract | 10.3389/fpsyt.2021.711835 | Google Scholar 34966296 PMC8710489

[B52] Shaun P. Chang C. (2019). PLINK 2.0. Available at: www.cog-genomics.org/plink/2.0/ . Google Scholar

[B53] Shen B. Yi X. Sun Y. Bi X. Du J. Zhang C. (2020). Proteomic and Metabolomic Characterization of COVID-19 Patient Sera. Cell 182 (1), 59–72.e15. 10.1016/j.cell.2020.05.032 PubMed Abstract | 10.1016/j.cell.2020.05.032 | Google Scholar 32492406 PMC7254001

[B54] Simeoni M. Cavinato T. Rodriguez D. Gatfield D. (2021). I(nsp1)ecting SARS-CoV-2-Ribosome Interactions. Commun. Biol. 4 (1), 715–5. 10.1038/s42003-021-02265-0 PubMed Abstract | 10.1038/s42003-021-02265-0 | Google Scholar 34112887 PMC8192748

[B55] Singh A. Shannon C. P. Gautier B. Rohart F. Vacher M. Tebbutt S. J. (2019). DIABLO: an Integrative Approach for Identifying Key Molecular Drivers from Multi-Omics Assays. Bioinformatics 35 (17), 3055–3062. 10.1093/bioinformatics/bty1054 PubMed Abstract | 10.1093/bioinformatics/bty1054 | Google Scholar 30657866 PMC6735831

[B56] Singh B. Kaur P. Patel P. Nabati C. Ayad S. Shamoon F. (2021). COVID-19 and Arterial Thrombosis: Report of 2 Cases. Radiol. Case Rep. 16 (7), 1603–1607. 10.1016/j.radcr.2021.04.033 PubMed Abstract | 10.1016/j.radcr.2021.04.033 | Google Scholar 33968287 PMC8084284

[B57] Stegle O. Parts L. Durbin R. Winn J. (2010). A Bayesian Framework to Account for Complex Non-Genetic Factors in Gene Expression Levels Greatly Increases Power in eQTL Studies. PLoS Comput. Biol. 6 (5), e1000770. 10.1371/journal.pcbi.1000770 PubMed Abstract | 10.1371/journal.pcbi.1000770 | Google Scholar 20463871 PMC2865505

[B58] Stephenson E. Reynolds G. Botting R. A. Calero-Nieto F. J. Morgan M. D. Tuong Z. K. (2021). Single-cell Multi-Omics Analysis of the Immune Response in COVID-19. Nat. Med. 27 (5), 904–916. 10.1038/s41591-021-01329-2 PubMed Abstract | 10.1038/s41591-021-01329-2 | Google Scholar 33879890 PMC8121667

[B59] Tan X. Wang Y. Han Y. Chang W. Su T. Hou J. (2013). Genetic Variation in the GSTM3 Promoter Confer Risk and Prognosis of Renal Cell Carcinoma by Reducing Gene Expression. Br. J. Cancer 109 (12), 3105–3115. 10.1038/bjc.2013.669 PubMed Abstract | 10.1038/bjc.2013.669 | Google Scholar 24157827 PMC3859948

[B60] Tani H. Imamachi N. Salam K. A. Mizutani R. Ijiri K. Irie T. (2012). Identification of Hundreds of Novel UPF1 Target Transcripts by Direct Determination of Whole Transcriptome Stability. RNA Biol. 9 (11), 1370–1379. 10.4161/rna.22360 PubMed Abstract | 10.4161/rna.22360 | Google Scholar 23064114 PMC3597577

[B61] Tay M. Z. Poh C. M. Rénia L. MacAry P. A. Ng L. F. P. (2020). The Trinity of COVID-19: Immunity, Inflammation and Intervention. Nat. Rev. Immunol. 20, 363–374. 10.1038/s41577-020-0311-8 PubMed Abstract | 10.1038/s41577-020-0311-8 | Google Scholar 32346093 PMC7187672

[B62] The Covid-19 Host Genetics Initiative (2020). The COVID-19 Host Genetics Initiative, a Global Initiative to Elucidate the Role of Host Genetic Factors in Susceptibility and Severity of the SARS-CoV-2 Virus Pandemic. Eur. J. Hum. Genet. 28 (6), 715–718. 10.1038/s41431-020-0636-6 PubMed Abstract | 10.1038/s41431-020-0636-6 | Google Scholar 32404885 PMC7220587

[B63] The Human Protein Atlas (2022). Available at: https://www.proteinatlas.org/ (Accessed March 13, 2022).

[B64] Tiwari R. Mishra A. R. Gupta A. Nayak D. (2021). Structural Similarity-Based Prediction of Host Factors Associated with SARS-CoV-2 Infection and Pathogenesis. J. Biomol. Struct. Dyn., 1–12. 10.1080/07391102.2021.1874532 10.1080/07391102.2021.1874532 | Google Scholar PMC7852281 33506741

[B65] Uhlén M. Fagerberg L. Hallström B. M. Lindskog C. Oksvold P. Mardinoglu A. (2015). Tissue-based Map of the Human Proteome. Science 347 (6220), 1260419. 10.1126/science.1260419 PubMed Abstract | 10.1126/science.1260419 | Google Scholar 25613900

[B66] Urbanek K. Torella D. Sheikh F. De Angelis A. Nurzynska D. Silvestri F. (2005). Myocardial Regeneration by Activation of Multipotent Cardiac Stem Cells in Ischemic Heart Failure. Proc. Natl. Acad. Sci. U.S.A. 102 (24), 8692–8697. 10.1073/pnas.0500169102 PubMed Abstract | 10.1073/pnas.0500169102 | Google Scholar 15932947 PMC1150816

[B67] Vardakas K. Z. Siempos I. I. Falagas M. E. (2007). Diabetes Mellitus as a Risk Factor for Nosocomial Pneumonia and Associated Mortality. Diabet. Med. 24 (10), 1168–1171. 10.1111/j.1464-5491.2007.02234.x PubMed Abstract | 10.1111/j.1464-5491.2007.02234.x | Google Scholar 17888136

[B68] Wang Y. Yang Y. Ren L. Shao Y. Tao W. Dai X. J. (2021). Preexisting Mental Disorders Increase the Risk of COVID-19 Infection and Associated Mortality. Front. Public Health 9, 684112. 10.3389/fpubh.2021.684112 PubMed Abstract | 10.3389/fpubh.2021.684112 | Google Scholar 34434913 PMC8381336

[B69] Wei J. Alfajaro M. M. DeWeirdt P. C. Hanna R. E. Lu-Culligan W. J. Cai W. L. (2021). Genome-wide CRISPR Screens Reveal Host Factors Critical for SARS-CoV-2 Infection. Cell 184 (1), 76–91. e13. 10.1016/j.cell.2020.10.028 PubMed Abstract | 10.1016/j.cell.2020.10.028 | Google Scholar 33147444 PMC7574718

[B70] Wu P. Chen D. Ding W. Wu P. Hou H. Bai Y. (2021). The Trans-omics Landscape of COVID-19. Nat. Commun. 12 (1), 4543. 10.1038/s41467-021-24482-1 PubMed Abstract | 10.1038/s41467-021-24482-1 | Google Scholar 34315889 PMC8316550

[B71] Zamakhchari M. F. Sima C. Sama K. Fine N. Glogauer M. Van Dyke T. E. (2016). Lack of P47phox in Akita Diabetic Mice Is Associated with Interstitial Pneumonia, Fibrosis, and Oral Inflammation. Am. J. Pathology 186 (3), 659–670. 10.1016/j.ajpath.2015.10.026 PubMed Abstract | 10.1016/j.ajpath.2015.10.026 | Google Scholar PMC4816692 26747235

