# Supplementary material for: Network Embedding Across Multiple Tissues and Data Modalities Elucidates the Context of Host Factors Important for COVID-19 Infection
Source: Front Genet. 2022 Jul 8;13:909714. doi: 10.3389/fgene.2022.909714 (PMC9315940; doi:10.3389/fgene.2022.909714)
Supplement: Supplementary file 1 [file DataSheet1.PDF]

Supplementary Material

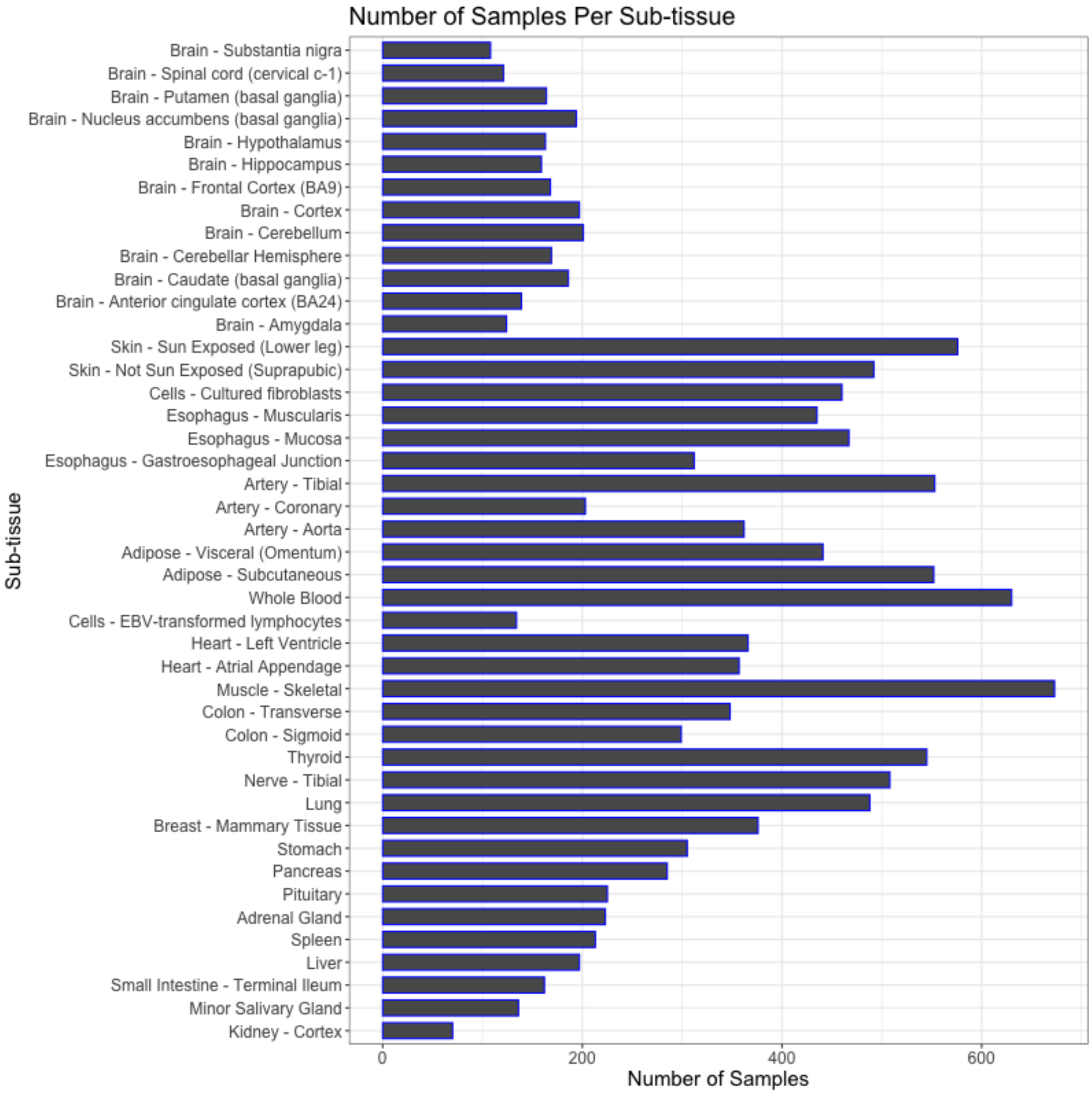

Supplementary Figure 1: Number of samples per sub-tissue type from the GTEx consortium used for the network inference

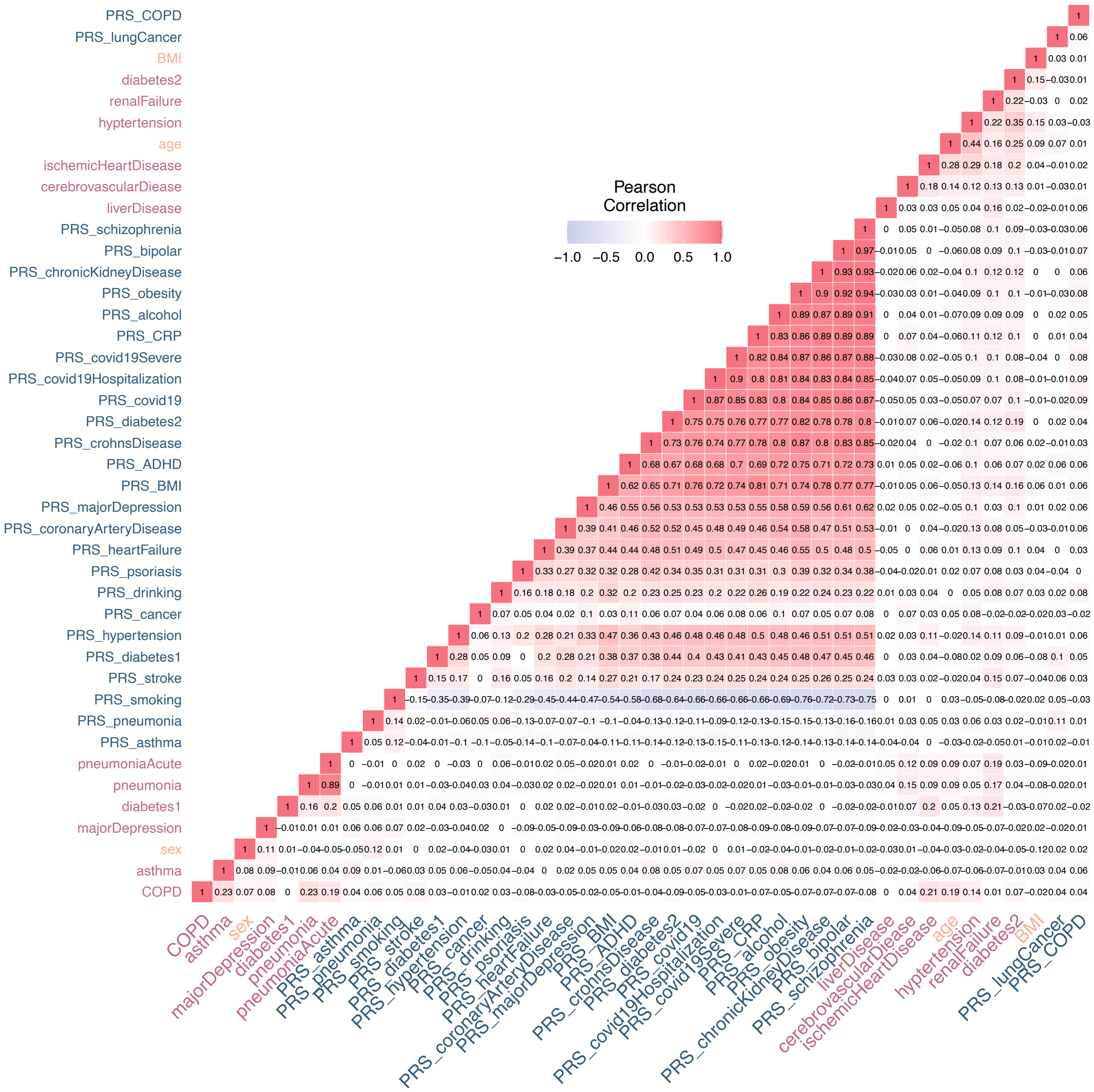

Supplementary Figure 2: Pearson correlation of different data modalities of Polygenic Risk Scores (PRS) in blue, comorbidities in purple and phenotypes in orange. Correlation was generally low across the different data modalities, making the integration of information very important in order to get a holistic view of ranging from genetic predisposition to actual comorbidity being influence by environmental effects. Higher correlations were observed between PRS

A

target	predictor	relation	mean_value	mean_rsq
RPL6	RPS9	prior_expr	0.0004894930	0.9127852
RPL6	RPS3	prior_expr	-0.0113043323	0.9127852
RPL6	RPS11	prior_expr	-0.0002571735	0.9127852
RPL6	RPL7	prior_expr	0.0129995783	0.9127852
RPL6	ILF2	prior_expr	-0.0004909220	0.9127852
RPL6	HNRNPU	prior_expr	0.0006112889	0.9127852
RPL6	FGF2	prior_expr	-0.0040520966	0.9127852
RPL6	Pituitary	expr_tissue	0.0182454607	0.9127852
RPL6	Muscle_Skeletal	expr_tissue	0.0002804242	0.9127852
RPL6	Lung	expr_tissue	0.0018316824	0.9127852
RPL6	PRS_pneumonia	expr_prs	0.0351627872	0.9127852
RPL6	PRS_drinking	expr_prs	0.0324385794	0.9127852
RPL6	PRS_diabetes2	expr_prs	0.0238945022	0.9127852
RPL6	PRS_asthma	expr_prs	0.0122384741	0.9127852
RPL6	PRS_COPD	expr_prs	0.0509888007	0.9127852
RPL6	diabetes2	expr_como	0.0042451691	0.9127852
RPL6	diabetes1	expr_como	0.0711155421	0.9127852
RPL6	PRS_pneumonia	expr_como	-0.0052523170	0.9127852
RPL6	age	expr_bio	-0.0008140422	0.9127852

B

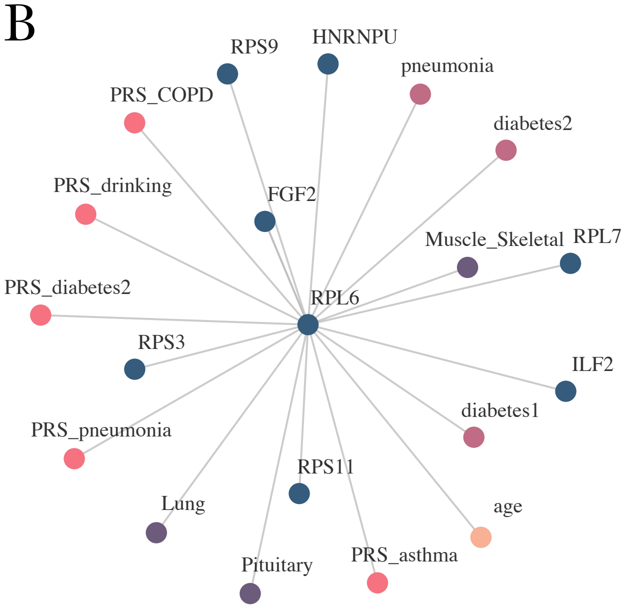

Supplementary Figure 3: Illustration of KiMONo's gene models A) from the sparse group lasso output with a R^2 of 0.912 and the mean beta coefficients over 30 runs of stability selection and B) the statistical associations displayed as edges between RPL6 and its explanatory variables from different modalities of genes, phenotypes, tissues, disease states and PRS

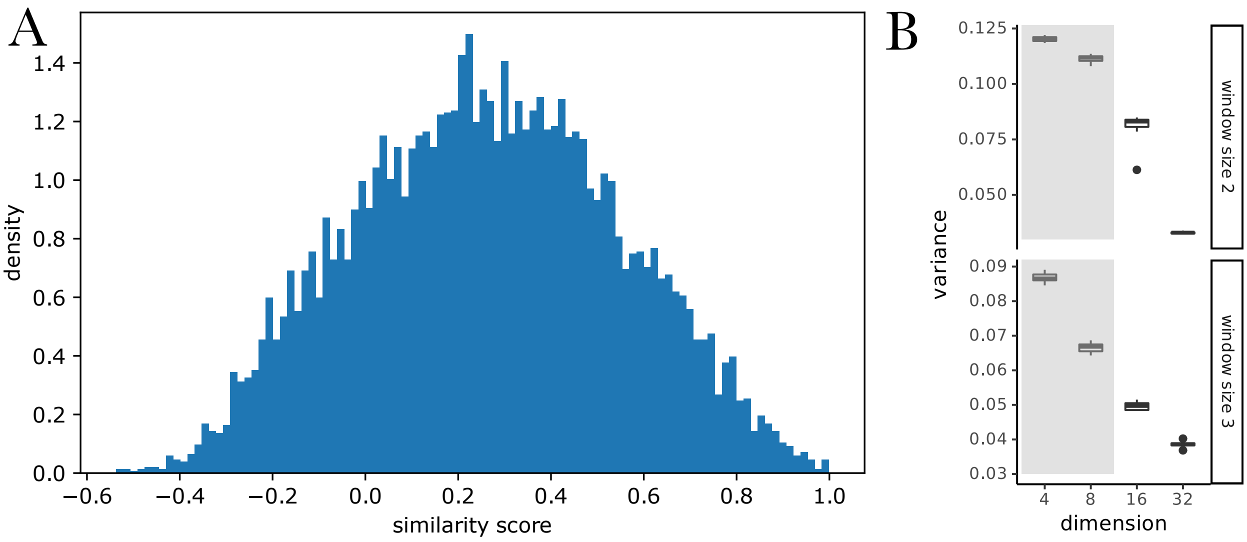

Supplementary Figure 4: Grid search of optimal parameters on small subnetwork of brain samples A) Similarity score distribution of one run with window size = 2 and embedding dimensionality = 16 between 10,000 random node pairs; variance = 0.080. B) variance of 10,000 random node pair similarities with window size [2,3] and dimensionality of embedding [4, 8,16, 32] over 10 embedding repetitions. Distributions that were not normally distributed are overlaid with gray background and thus discarded for parameter selection.

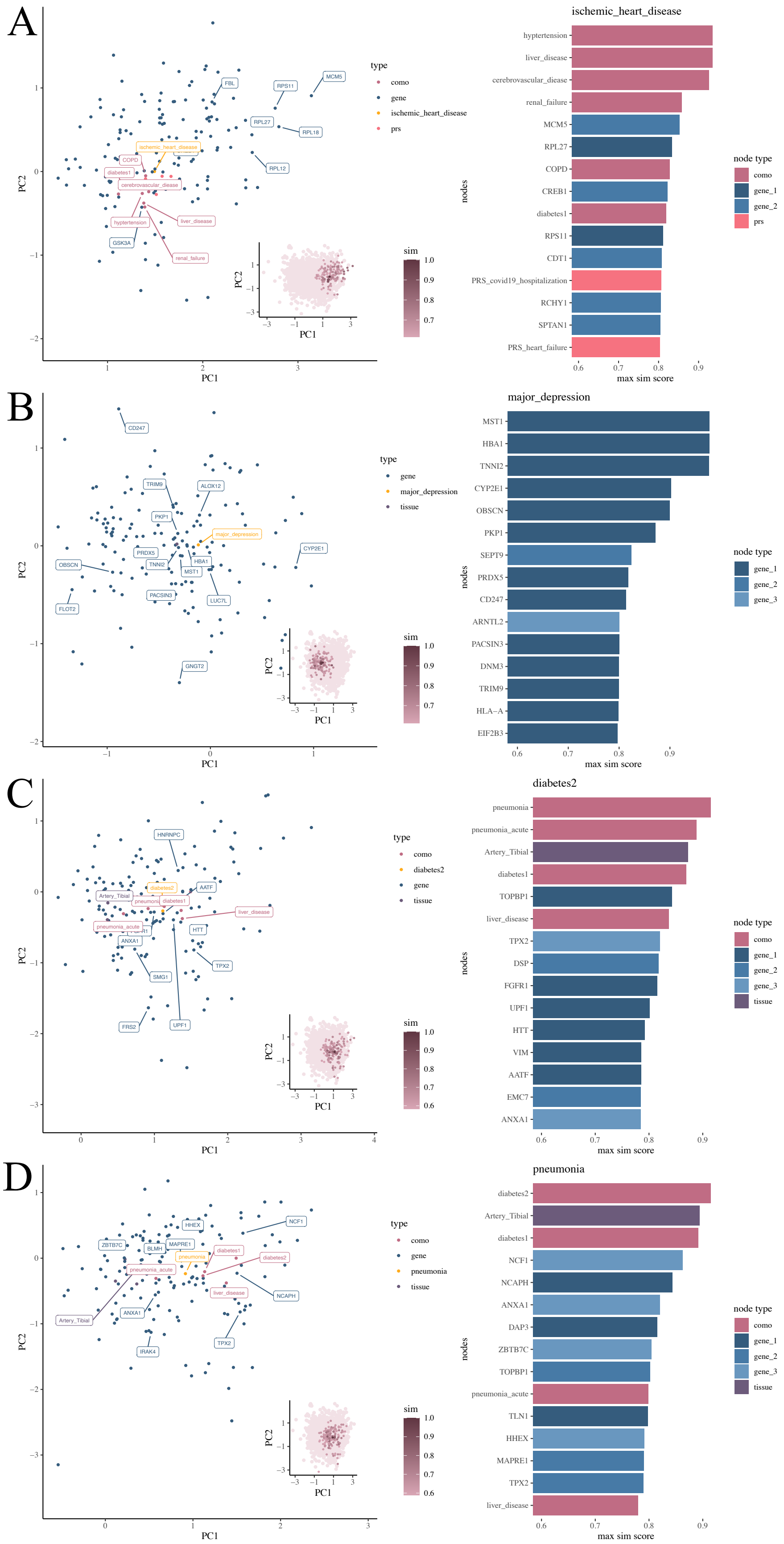
A
type
como
gene
ischemic_heart_disease
prs
PC1
PC2
sim
1.0
0.9
0.8
0.7
ischemic_heart_disease
hyptertension
liver_disease
cerebrovascular_disease
renal_failure
MCM5
RPL27
COPD
CREB1
diabetes1
RPS11
CDT1
PRS_covid19_hospitalization
RCHY1
SPTAN1
PRS_heart_failure
nodes
max sim score
node type
como
gene_1
gene_2
prs
B
type
gene
major_depression
tissue
major_depression
MST1
HBA1
TNNI2
CYP2E1
OBSCN
PKP1
SEPT9
PRDX5
CD247
ARNTL2
PACSIN3
DNM3
TRIM9
HLA−A
EIF2B3
node type
gene_1
gene_2
gene_3
C
type
como
diabetes2
gene
tissue
diabetes2
pneumonia
pneumonia_acute
Artery_Tibial
diabetes1
TOPBP1
liver_disease
TPX2
DSP
FGFR1
UPF1
HTT
VIM
AATF
EMC7
ANXA1
node type
como
gene_1
gene_2
gene_3
tissue
D
type
como
gene
pneumonia
tissue
pneumonia
diabetes2
Artery_Tibial
diabetes1
NCF1
NCAPH
ANXA1
DAP3
ZBTB7C
TOPBP1
pneumonia_acute
TLN1
HHEX
MAPRE1
TPX2
liver_disease
node type
como
gene_1
gene_2
gene_3
tissue

Supplementary Figure 5: Embedding space of comorbidities A) ischemic heart disease, B) major depression, C) Diabetes Type II and D) pneumonia

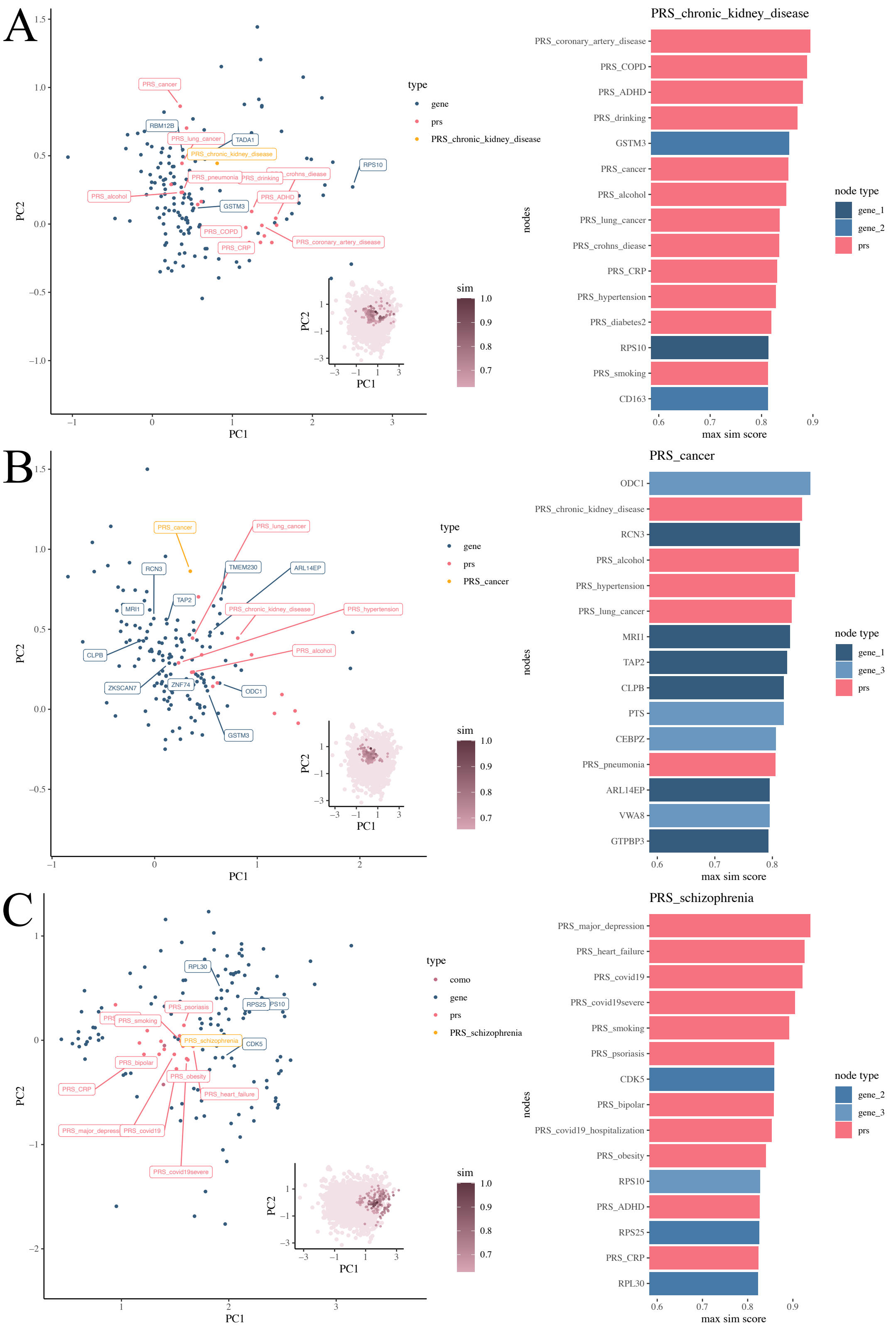

Supplementary Figure 6: Embedding space of PRS for A) chronic kidney disease, B) cancer and C) schizophrenia
